# The SCD1 inhibitor aramchol interacts with regorafenib to kill GI tumor cells *in vitro* and *in vivo*

**DOI:** 10.18632/oncotarget.28762

**Published:** 2025-08-19

**Authors:** Laurence Booth, Michael R. Booth, Jane L. Roberts, Yang Yue, Emily Kinsey, Andrew Poklepovic, David Boone, L. Ashley Cowhart, Allen Baharaff, Paul Dent

**Affiliations:** ^1^Department of Biochemistry and Molecular Biology, Virginia Commonwealth University, Richmond, VA 23298, USA; ^2^Department of Medicine, Virginia Commonwealth University, Richmond, VA 23298, USA; ^3^Galmed Pharmaceuticals, Tel Aviv 6578317, Israel; ^4^Indiana University School of Medicine and University of Notre Dame, South Bend, IN 46556, USA; ^*^Equal co-authors

**Keywords:** macroautophagy, flux, ER stress, aramchol, regorafenib

## Abstract

The anti-tumor actions of the Stearoyl-CoA desaturase (SCD1) inhibitor aramchol in tumor cells remains poorly understood. Aramchol interacted with the multi-kinase inhibitors sorafenib, regorafenib or lenvatinib, to kill GI tumor cells, with regorafenib exhibiting the greatest effect. In HCT116 cells homozygous for the autophagy-regulatory protein ATG16L1 T300, aramchol and regorafenib interacted to activate ATM and the AMPK and to inactivate mTORC1 and mTORC2. As a single agent, regorafenib inactivated eIF2α and it combined with aramchol to elevate GRP78 expression. In HCT116 cells expressing the ATG16L1 A300 isoform the drug-induced dephosphorylation of mTORC1 S2448 and mTORC2 S2481 and the increased phosphorylation of eIF2α S51 were significantly lower than in T300 cells. In cells expressing ATG16L1 T300, but not A300, regorafenib and/or the drug combination inactivated AKT, ERK1/2 and p70 S6K. Regorafenib and aramchol interacted to cause formation of autophagosomes which was significantly greater in cells expressing ATG16L1 T300. Aramchol as a single agent did not stimulate autophagic flux but further enhanced both flux and autolysosome formation caused by regorafenib. Knock down of Beclin1 reduced the lethality of regorafenib and aramchol as single agents and when combined whereas knock down of LAMP2 or BID did not reduce killing caused by aramchol as a single agent but did reduce the lethality of regorafenib alone and regorafenib plus aramchol. *In vivo* using the HuH7 adult hepatoma cell line, regorafenib and aramchol interacted to suppress tumor growth without normal tissue toxicities.

## INTRODUCTION

The Stearoyl-CoA desaturase (SCD1) inhibitor aramchol was initially developed for patients with Metabolically Associated steatohepatitis (MASH) [1]. The drug concentrates in the liver over other tissues and its tissue concentration within the liver is over 100 μM at steady state [2]. Aramchol both enzymatically inhibits SCD1 and over time reduces SCD1 protein levels [3, 4]. The loss of SCD1 initially results in enhanced fatty acid beta-oxidation followed by increased cellular glutathione levels, thus stabilizing and normalizing the redox status of cells [5]. From these actions, aramchol reduces liver fibrosis and improves the performance status of MASH patients [6].

Compared to non-transformed cells, cancer cells generally generate much larger amounts of reactive oxygen species (ROS) which play key roles in maintaining tumor cell biology and the malignant phenotype. Decreased ROS production or by therapeutically quenching ROS in a tumor cell ultimately leads to cell death. In non-transformed cells, aramchol causes increased glutathione levels which in a tumor cell will lower ROS levels. In addition, inhibition of SCD1 reduces the expression of multiple proteins essential for glucose utilization leading to mitochondrial dysfunction [5]. Collectively, these findings argue aramchol may have utility as an anti-cancer drug.

Precision drug development should include understanding genetic differences in any targeted population. In the past, major studies of health disparities comparing European Americans and African Americans in The United States have been based on access to health care and diet and it is known that very few genetic differences can be demarcated to those from a specific geographic origin. The autophagosome-regulatory gene ATG16L1 has two isoforms [7, 8]. In those of northern European ancestry, the isoform ATG16L1 A300 is most prevalent whereas for those of African ancestry, and to a lesser extent in other parts of the world, the most common isoform is ATG16L1 T300. This difference has “real world” relevance as people homozygous for ATG16L1 A300/A300 are significantly more likely than those with ATG16L1 T300/T300 to present with Crohn’s Disease [9]. One putative mechanism in the development of Crohn’s, which we have confirmed previously in oncological settings, is that T300/T300 cells are significantly more capable than A300/A300 cells at the autophagic digestion and elimination of proteins and immunogenic antigens [10, 11].

Multi-kinase inhibitors as anti-cancer therapeutics have been under development for over thirty years. One of the first drugs developed in this class was sorafenib (Nexavar^®^), shortly followed by a derivative of the agent, regorafenib (Stivarga^®^) which has an additional fluorine atom in its structure [12, 13]. As their names suggest, both drugs were developed to inhibit the RAF-1 and B-RAF kinases, upstream components of the ERK1/2 pathway which was a pathway known to signal causing tumor cell growth and resistance to older cytotoxic chemotherapies. Subsequently, both drugs were shown to inhibit receptor tyrosine kinases and for sorafenib but not regorafenib, to inhibit the ATPase activities of chaperone proteins [14–16]. Regorafenib and sorafenib are both approved for the treatment of liver cancer, and additionally regorafenib for colorectal cancers.

One problematic issue in cancer developmental therapeutics studies with novel drug combinations is defining the correct concentrations of the agents to be used for *in vitro* assays. Studies by our group over the past ~20 years have used the safely achievable maximal plasma concentration (C max) and protein binding of an agent as guidelines for its concentration range to be used for *in vitro* assays [12–14]. For example, the C max of sorafenib is approximately 13 μM, however because of protein binding, the probable “free” sorafenib concentration will be at or below 2 μM. Similarly, with regorafenib, the probable free concentration of this agent will be less than 1 μM; in our studies, we have generally used sorafenib (2.0 μM) and regorafenib (0.5 μM) [15, 16]. Other groups who have investigated the interactions of aramchol with multi-kinase inhibitors used *in vitro* concentrations of up to 10 μM of the kinase inhibitor drugs, casting doubt on whether such findings can be reliably used to support translation into the clinic [17].

The purpose of our studies was to define the interactions, if any, between aramchol and a multi-kinase inhibitor in GI tumor cells at clinically relevant drug concentrations and to define the responses of isogenic cells homozygous for ATG16L1 T300 or ATG16L1 A300 [18–23]. We determined in GI tumor cells that regorafenib interacted with aramchol to promote tumor cell killing. T300/T300 tumor cells were more efficaciously killed by the aramchol plus regorafenib combination than A300/A300 cells. Our *in vitro* findings were mirrored by *in vivo* findings in a mouse model of hepatoma.

## RESULTS

**Figure 1:**
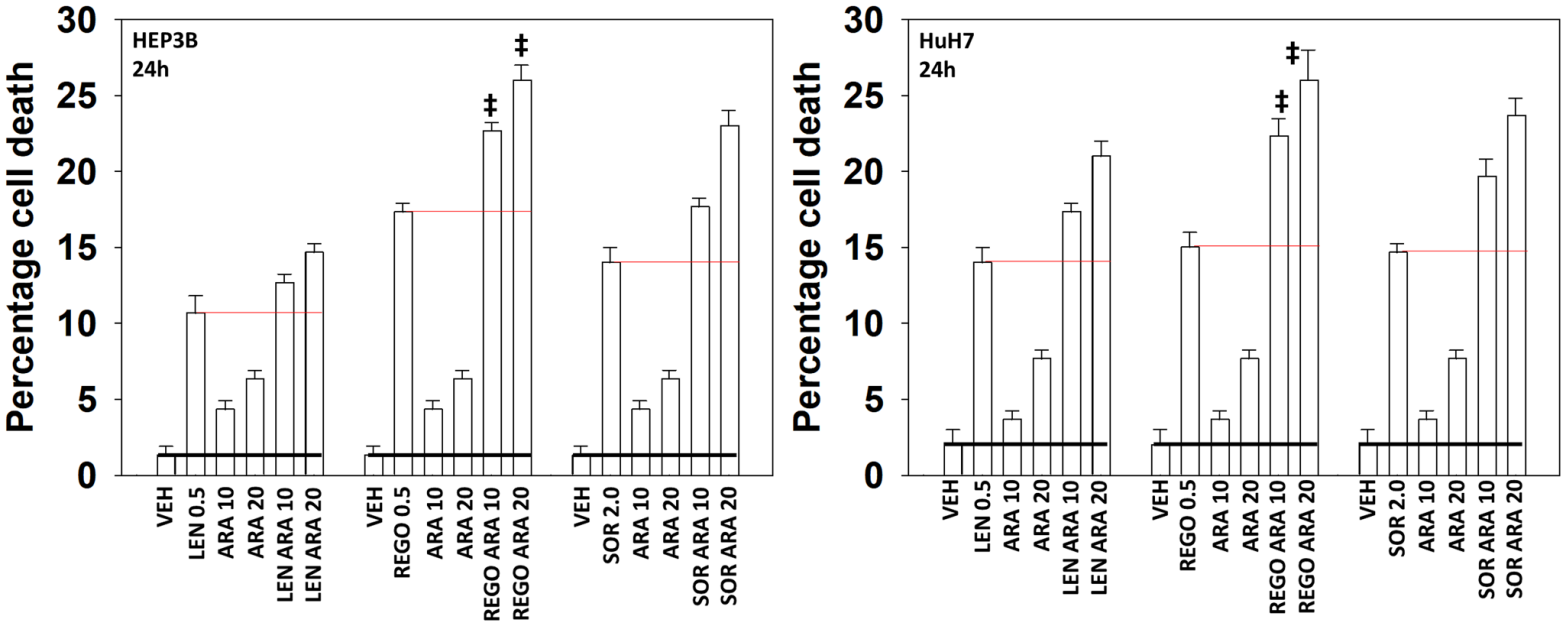
Aramchol and regorafenib interact to kill hepatoma cells. HEP3B and HuH7 cells were treated with vehicle control, aramchol (10 μM, 20 μM), regorafenib (0.5 μM), lenvatinib (0.5 μM), sorafenib (2.0 μM) or the drugs combined as indicated for 24 h. Floating and attached cells from three independent studies were collected and the percentage viability determined using trypan blue exclusion assays (±SD). ^‡^ *p* < 0.05 greater than corresponding values in cells treated with either sorafenib or lenvatinib.

Our initial studies sought to determine, between three FDA-approved multi-kinase inhibitors, which would interact with aramchol in the most efficacious fashion to kill hepatoma cells. Three kinase inhibitors were tested, sorafenib, regorafenib and the more recently developed lenvatinib, with hopes from our medical colleagues that the newer readily available drug lenvatinib would prove to be the most useful (Figure 1). In both HEP3B and HuH7 hepatoma cells, however, regorafenib exhibited the most significant toxic interaction with aramchol compared to either sorafenib or lenvatinib.

We next performed side-by-side experiments comparing the changes in cell signaling caused by regorafenib, aramchol and the drugs combined in ATG16L1 T300/T300 and ATG16L1 A300/A300 HCT116 colorectal cancer cells and in HuH7 hepatoma cells (Supplementary Tables 1–4).

From our analyses, several important pieces of information were gleaned. Regardless of ATG16L1 isoform expression, the drugs alone or in combination activated ATM and the AMPK to a similar extent. However, the drugs inactivated mTORC1 and mTORC2 in T300/T300 cells to a significantly greater extent than in A300/A300 cells. This was mirrored in changes to the phosphorylation of downstream targets such as ULK1 S317/S757. In T300/T300 cells, regorafenib caused the inactivation of ERK1/2 and p70 S6K. Regorafenib as a single agent increased endoplasmic reticulum (ER) stress signaling as judged by enhanced phosphorylation of PERK and eIF2α, and by elevated GRP78 expression. The amount of ERK1/2 and p70 S6K inactivation and the enhanced ER stress signaling induced by the drugs was significantly less in A300/A300 cells.

HCT116 cells express a mutant KRAS protein, a mutant p110 PI3K protein and both wild type p53 and PTEN. Growth factor receptors of the ERBB family lie upstream of RAS proteins and these receptors can both promote GTP loading in wild type RAS proteins and maintain GTP levels in mutant RAS proteins that have residual GTPase activity [24, 25]. In T300/T300 cells, regorafenib as a single agent reduced ERBB1 phosphorylation and it interacted with aramchol to inactivate ERBB2 and ERBB3. In A300/A300 cells these effects were reduced or absent. In HuH7 hepatoma cells, the drug-induced alterations in protein phosphorylation and protein expression were intermediate between those observed T300/T300 and A300/A300 HCT116 cells.

**Figure 2:**
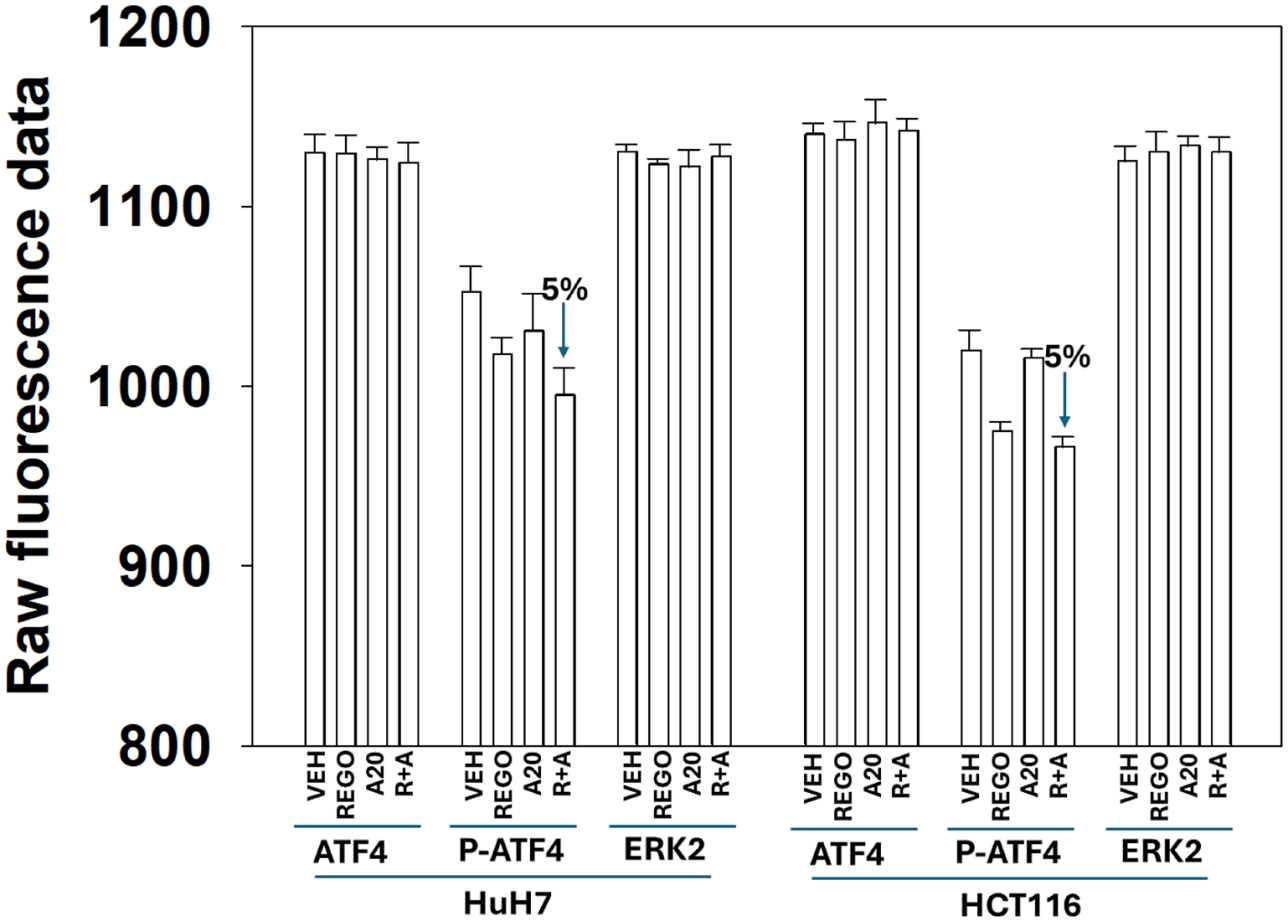
ATF4 expression and ATF4 phosphorylation are not significantly altered after exposure to aramchol and regorafenib. HCT116 ATG16L1 T300/T300 and HuH7 adult hepatoma cells were treated with drugs for 4 h. Cells were fixed in place an in-cell immunostaining performed to detect the protein levels of each protein, as indicated. Parallel staining for ERK2 was performed as a loading control (*n* = 3 independent assessments, ±SD).

Although we observed enhanced phosphorylation of PERK and eIF2α we did not see a corresponding increase, downstream, in the expression of CHOP. The transcription factor ATF4 lies between eIF2α and CHOP in this ER stress sensing pathway. Drug treatments neither enhanced ATF4 expression nor did they significantly alter ATF4 phosphorylation (Figure 2). Comparing the expression of ER-stress regulatory chaperone proteins in T300/T300 and A300/A300 cells we discovered that A300/A300 cells expressed lower total amounts and plasma membrane-localized GRP78 whereas the levels of HSP27, HSP70 and HSP90 were identical (Supplementary Figures 1 and 2). Thus, from our data sets in the T300/A300 HCT116 cells, where significant changes in protein expression or protein phosphorylation were observed in T300/T300 cells, the changes we saw in A300/A300 cells were either significantly reduced or abolished.

We next determined the pathway of signaling from ATM to autophagosome formation. In cells with ATM knocked down, drug exposure did not enhance AMPKα T172 phosphorylation and did not reduce mTORC1 S2448 phosphorylation (Table 1, upper). In cells with AMPKα knocked down, drug exposure did not cause dephosphorylation of mTORC1 S2448 nor increased phosphorylation of ATG13 S318, the gatekeeper phosphorylation event for autophagosome formation (Table 1, lower) [24, 26]. We conclude that cells homozygous for T300/T300 respond more strongly to regorafenib plus aramchol across multiple signaling parameters and that they are more readily killed by regorafenib plus aramchol. The reason for the lack of enhanced ATF4 and CHOP expression after ER stress signaling from PERK and eIF2α remains unresolved.

**Table 1:** ATM regulates AMPK and mTOR signaling and ATG13 S318 phosphorylation

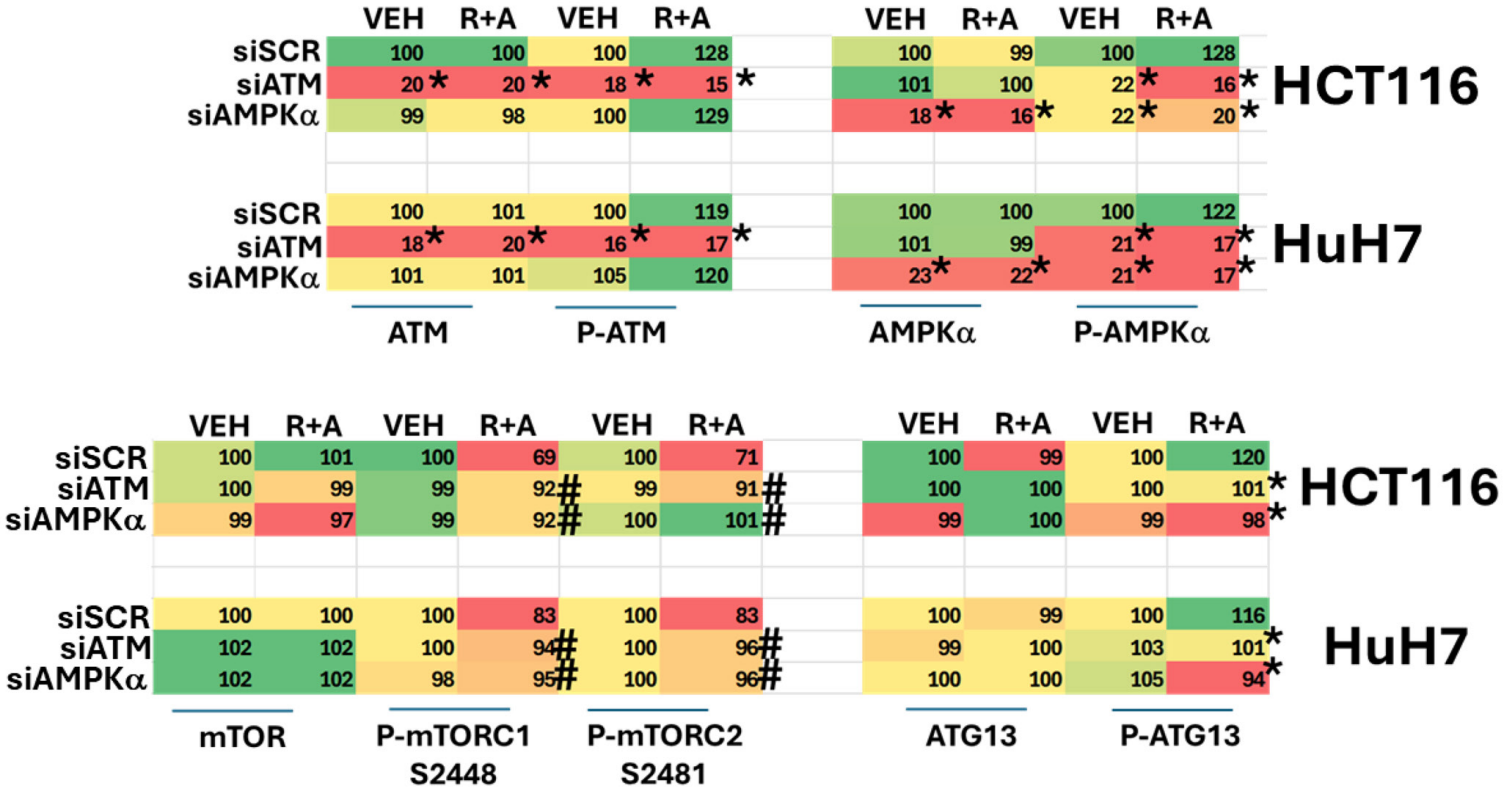

HCT116 ATG16L1 T300/T300 cells were transfected with a scrambled siRNA control (siSCR) or with validated siRNA molecules to knock down the expression of ATM or AMPKα. The percentage alteration in expression/phosphorylation caused by the drugs was determined from three independent replicates (±SD). ^*^ *p* < 0.05 less than vehicle control; ^#^ *p* < 0.05 greater than vehicle control value.

**Figure 3:**
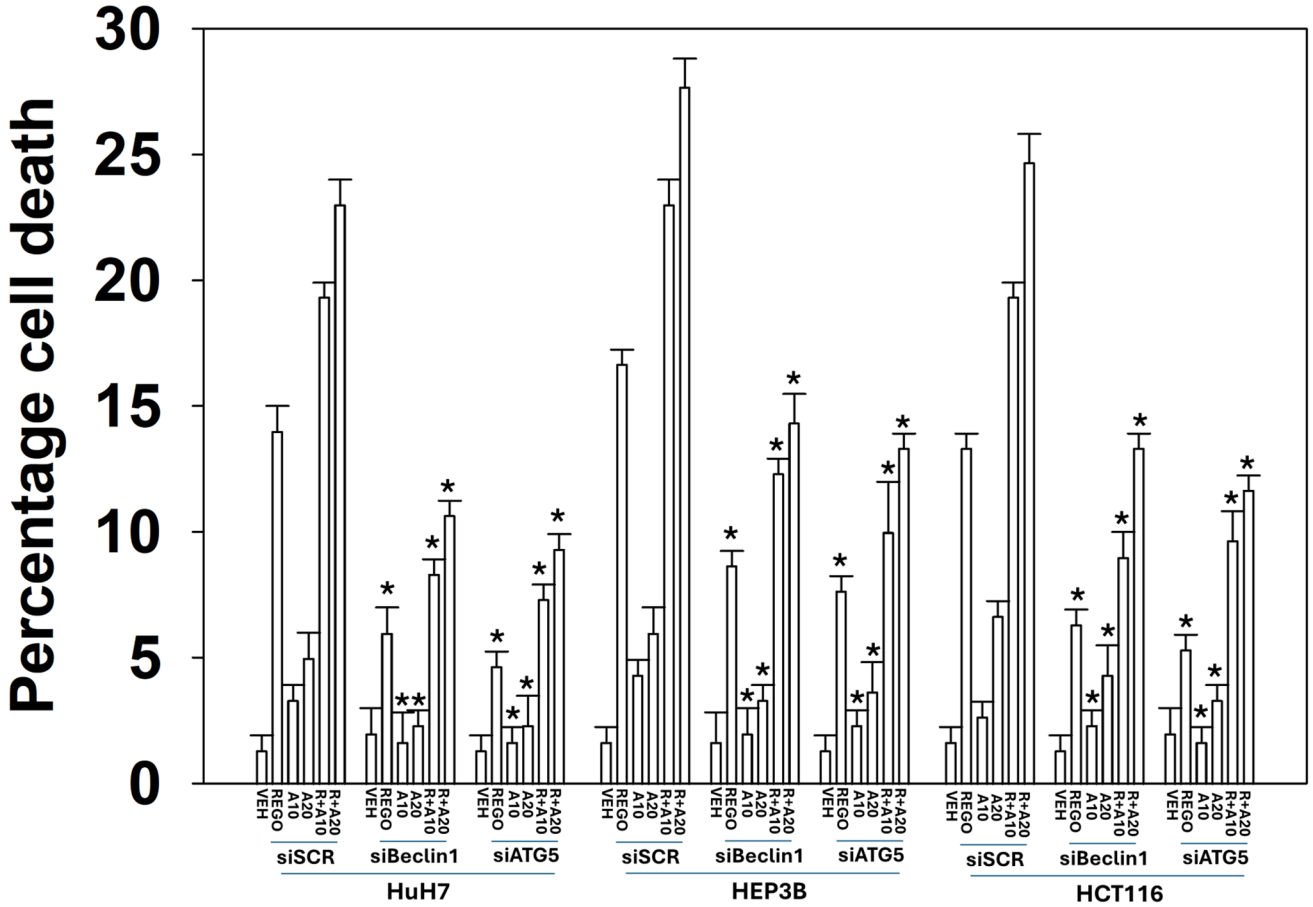
The lethal effects of the aramchol plus regorafenib combination require autophagosome formation. HuH7, HEP3B and wild type HCT116 T300/T300 cells were transfected with a scrambled siRNA or with validated siRNA molecules to knock down the expression of Beclin1 or ATG5. Floating and attached cells from three independent studies were collected and viability determined using trypan blue exclusion assays (±SD). ^*^ *p* < 0.05 less than corresponding value in siSCR transfected cells.

Data from prior studies using multi-kinase inhibitors demonstrated that the increased levels of autophagosomes after treatment were dependent on the pathway ATM-AMPK-ULK1/mTOR – ATG13 [24, 26]. In our experience, anti-cancer drug combinations using regorafenib utilize macroautophagy as a component of the killing mechanism. Hence, we next determined whether the combination of regorafenib with aramchol required autophagy to kill tumor cells. Knock down of the essential autophagosome-formation regulatory proteins Beclin1 or ATG5 significantly reduced the lethality of regorafenib or aramchol as single agents, and when they were combined (Figure 3). Knock down of Beclin1 or ATG5 did not abolish the toxic interaction between regorafenib and aramchol.

**Figure 4:**
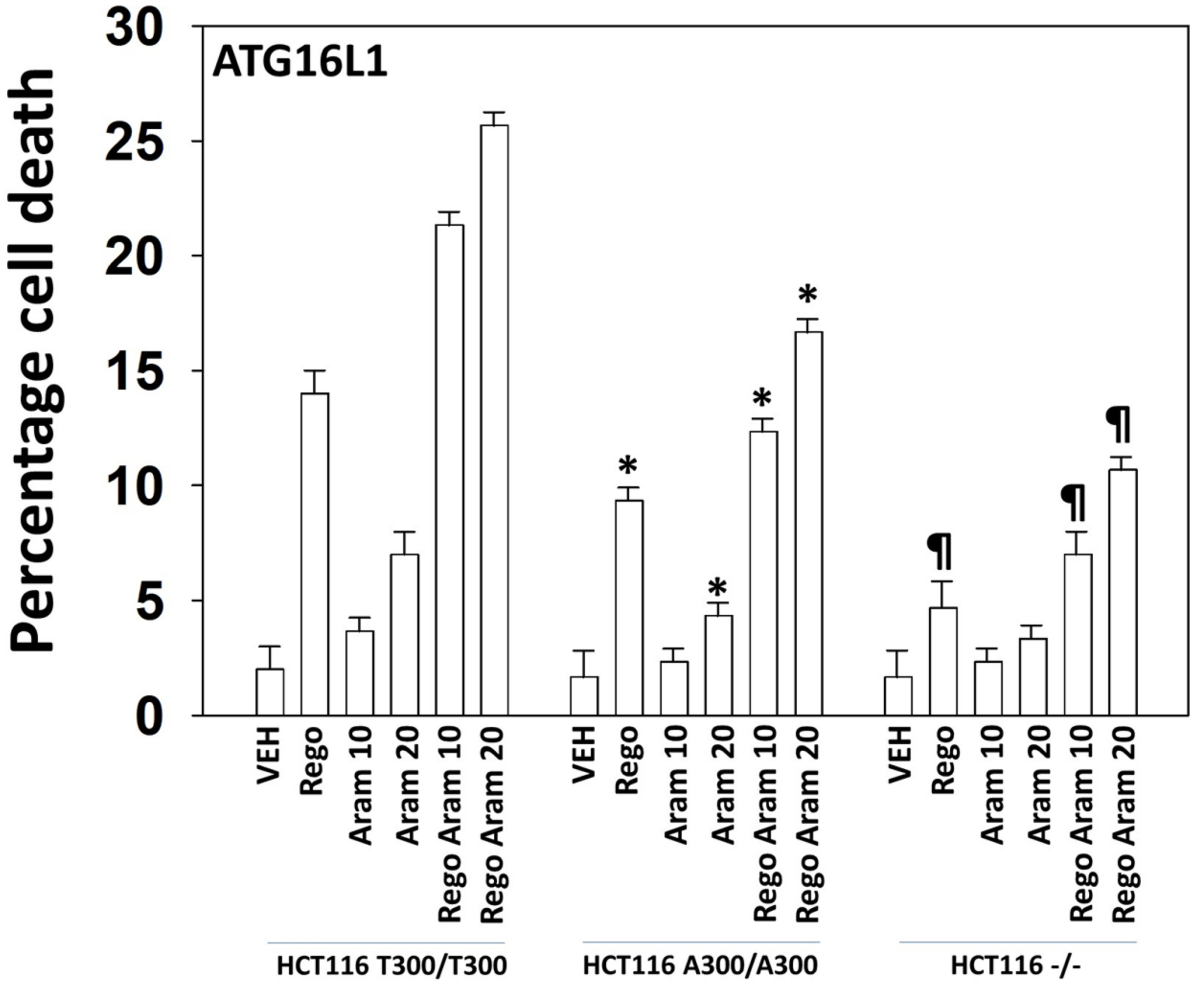
Aramchol and regorafenib, alone or in combination, preferentially kill HCT116 ATG16L1 T300/T300 cells compared to HCT116 ATG16L1 A300/A300 cells. HCT116 ATG16L1 T300/T300, HCT116 ATG16L1 A300/A300 and HCT116 ATG16L1 null cells were treated with drugs for 24 h. Floating and attached cells from three independent studies were collected and viability determined using trypan blue exclusion assays (±SD). ^*^ *p* < 0.05 less than corresponding value in T300/T300 cells; ^¶^ *p* < 0.05 less than corresponding value in HCT116 ATG16L1 A300/A300 cells.

We have previously published that multi-kinase inhibitors alone or in combination with other agents more effectively kill HCT116 cells homozygous for T300/T300 compared to isogenic cells expressing A300/A300 and that this was due to reduced autophagosome formation and autophagic flux [24]. Thus, we next made use of these isogenic HCT116 cell lines to link changes in cell signaling caused by the drugs to determine whether A300/A300 cells were less effectively killed by the regorafenib plus aramchol combination. A300/A300 cells were significantly more resistant to regorafenib or aramchol as single agents, and when they were combined compared to T300/T300 cells (Figure 4). In isogenic HCT116 cells that did not express ATG16L1 and are almost incapable of forming autophagosomes, the lethality of regorafenib or aramchol as single agents, and when they were combined, was significantly lower than that observed in ATG16L1 A300/A300 cells.

**Figure 5:**
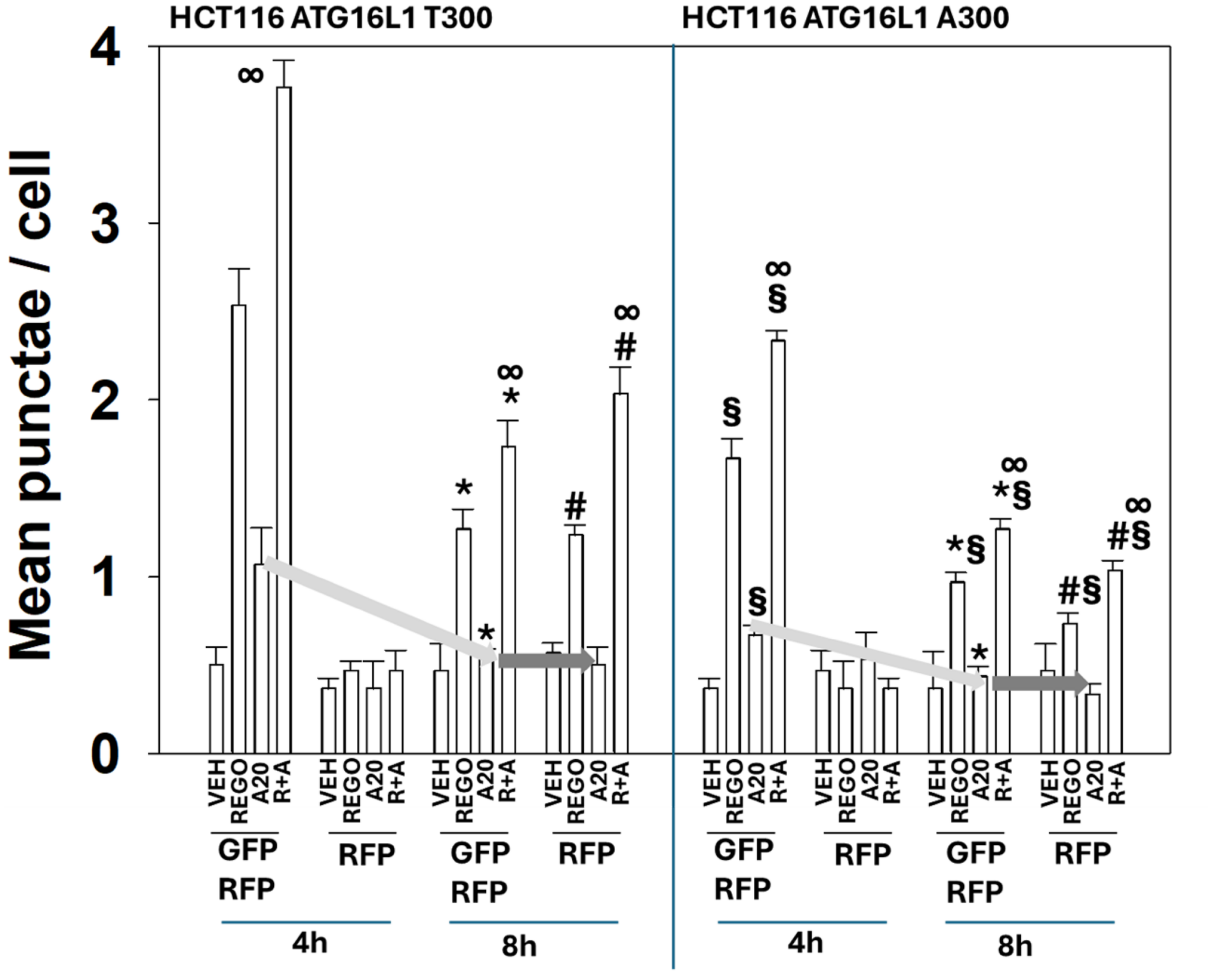
Aramchol increases autophagosome formation but not flux and yet it promotes autolysosome formation in the presence of regorafenib. T300/T300 and A300/A300 cells were transfected to express LC3-GFP-RFP. Cells were treated with drugs for 4 h and 8 h. At each time point, the mean numbers of intense (GFP + RFP) and (RFP alone) vesicles from 100 randomly selected cells were determined. (*n* = 3 ± SD). ^∞^ *p* < 0.05 greater than regorafenib alone value; ^*^ *p* < 0.05 less than corresponding value at the 4 h time point; ^§^ *p* < 0.05 less than corresponding values in T300 cells; ^#^ *p* < 0.05 greater than corresponding values at the 4 h time point.

To measure macroautophagy and autophagic flux, we made use of a plasmid that encodes for expression of a fusion protein, LC3-GFP-RFP. In the early autophagosome, both GFP and RFP fluoresce, and yellow vesicles are observed. After the fusion of the autophagosome with a lysosome, followed by its acidification, an autolysosome can be detected examining the numbers of red vesicles, as GFP fluorescence is quenched under acidic conditions. In Figure 5, in T300/T300 cells, both regorafenib and aramchol initially increased the numbers of intense yellow staining (GFP plus RFP) autophagosomes. Over time, the numbers of autophagosomes declined, and the numbers of red staining (RFP only) autolysosomes increased. This reduction in (GFP plus RFP) vesicles over time and an increase in (RFP only) vesicles is indicative of “autophagic flux.” Similar macroautophagy data, albeit with less amplitude, was observed in A300/A300 cells.

In both T300/T300 and A300/A300 cells two interrelated phenomena were observed that in over 15 years of studying macroautophagy, we had not previously seen. Unexpectedly, whilst treatment of cells with regorafenib as a single agent increased the numbers of (GFP plus RFP) vesicles that declined over time followed by the numbers of (RFP only) vesicles increasing, we did not observe this for aramchol. Aramchol as a single agent increased the numbers of (GFP plus RFP) vesicles that declined over time, but we did not see a corresponding increase in the numbers of (RFP only) vesicles. This implies that aramchol can stimulate the “first half” of macroautophagy, i.e., autophagosome formation, but as a single agent, it cannot enhance autophagic flux. However, although as a single agent aramchol did not enhance autophagic flux, it *did* enhance flux and autolysosome formation in the presence of regorafenib.

**Figure 6:**
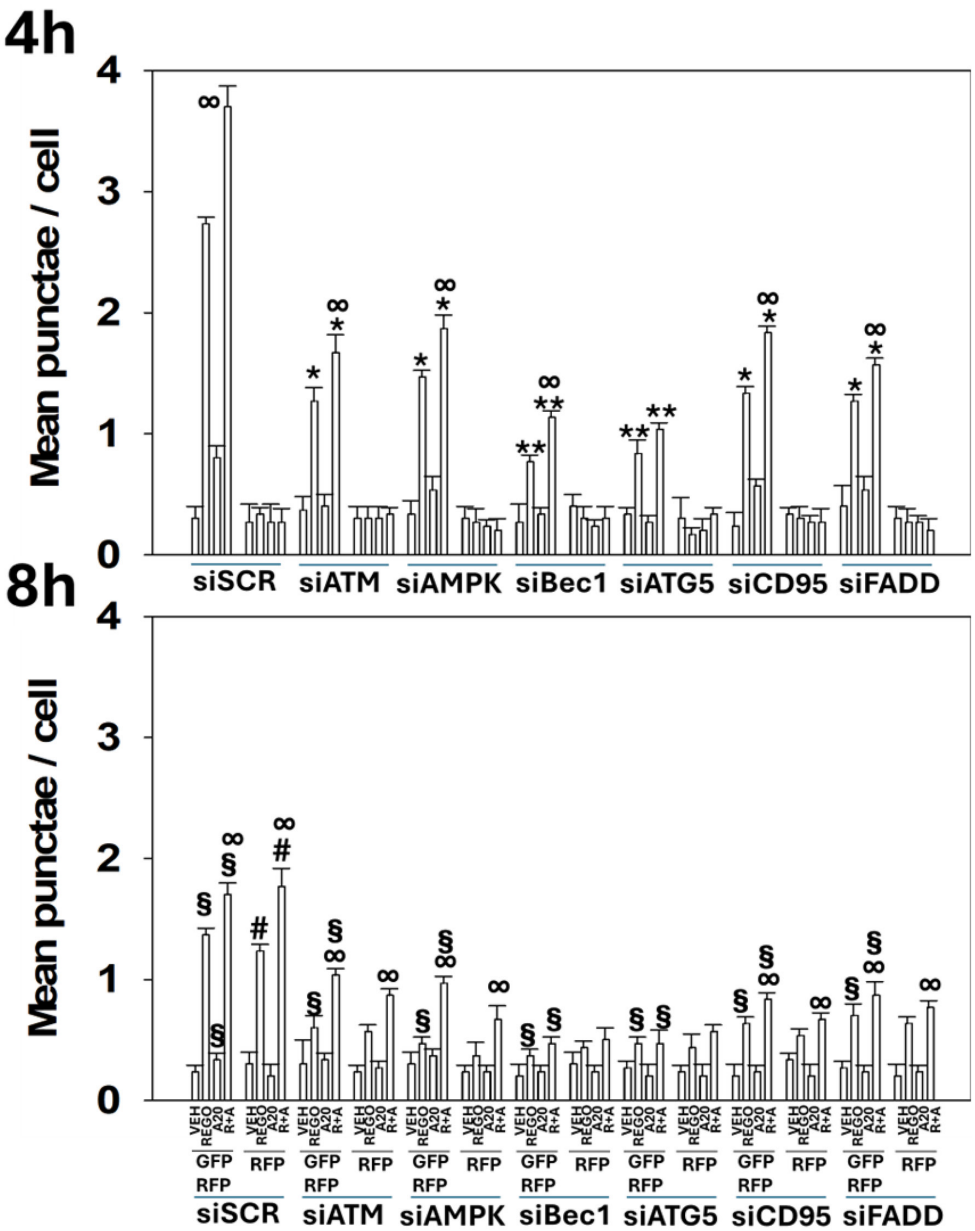
Aramchol plus regorafenib exposure causes autophagosome and autolysosome formation that is in part dependent on the actions of ATM, AMPK, CD95 and FADD. T300/T300 cells were transfected with a plasmid to express LC3-GFP-RFP and with a scrambled siRNA control (siSCR) or with siRNA molecules to knock down protein expression. Cells were treated with drugs for 4 h and 8 h. At each time point, the mean numbers of intense (GFP + RFP) and (RFP alone) vesicles from 100 randomly selected cells were determined. (*n* = 3 ± SD). ^∞^ *p* < 0.05 greater than regorafenib alone value; ^*^ *p* < 0.05 less than corresponding value at the 4 h time point; ^§^ *p* < 0.05 less than corresponding values in T300 cells; ^#^ *p* < 0.05 greater than corresponding values at the 4 h time point.

**Figure 7:**
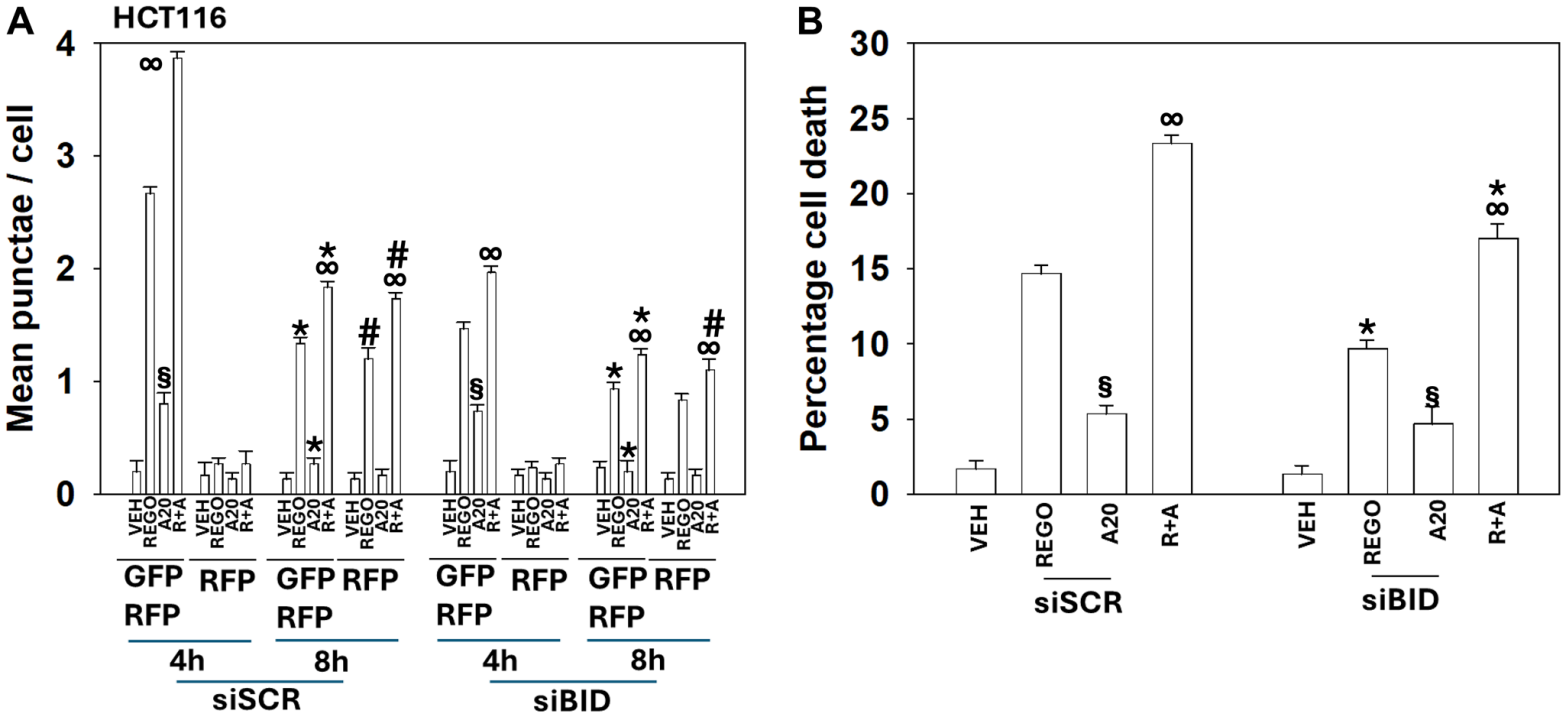
In HCT116 cells knock down of BID reduces aramchol-induced autophagic flux and tumor cell death. (**A**) T300/T300 cells were transfected to express LC3-GFP-RFP and siRNA transfected to knock down BID expression. Cells were treated with drugs for 4 h and 8 h. The mean numbers of intense (GFP + RFP) and (RFP alone) vesicles from 100 randomly selected cells were determined. (*n* = 3 ± SD). ^∞^ *p* < 0.05 greater than regorafenib alone value; ^*^ *p* < 0.05 less than corresponding value at the 4 h time point; ^#^ *p* < 0.05 greater than corresponding values at the 4 h time point; ^§^ *p* > 0.05 comparing vesicle formation in siSCR and siBID cells. (**B**) T300/T300 cells were transfected to knock down expression of BID. Cells were treated with drugs for 24 h. The percentage viability was determined using trypan blue exclusion assays (±SD). ^∞^ *p* < 0.05 greater than regorafenib alone value; ^*^ *p* < 0.05 less than corresponding value in siSCR cells; ^§^ *p* > 0.05 comparing vesicle formation in siSCR and siBID cells.

**Figure 8:**
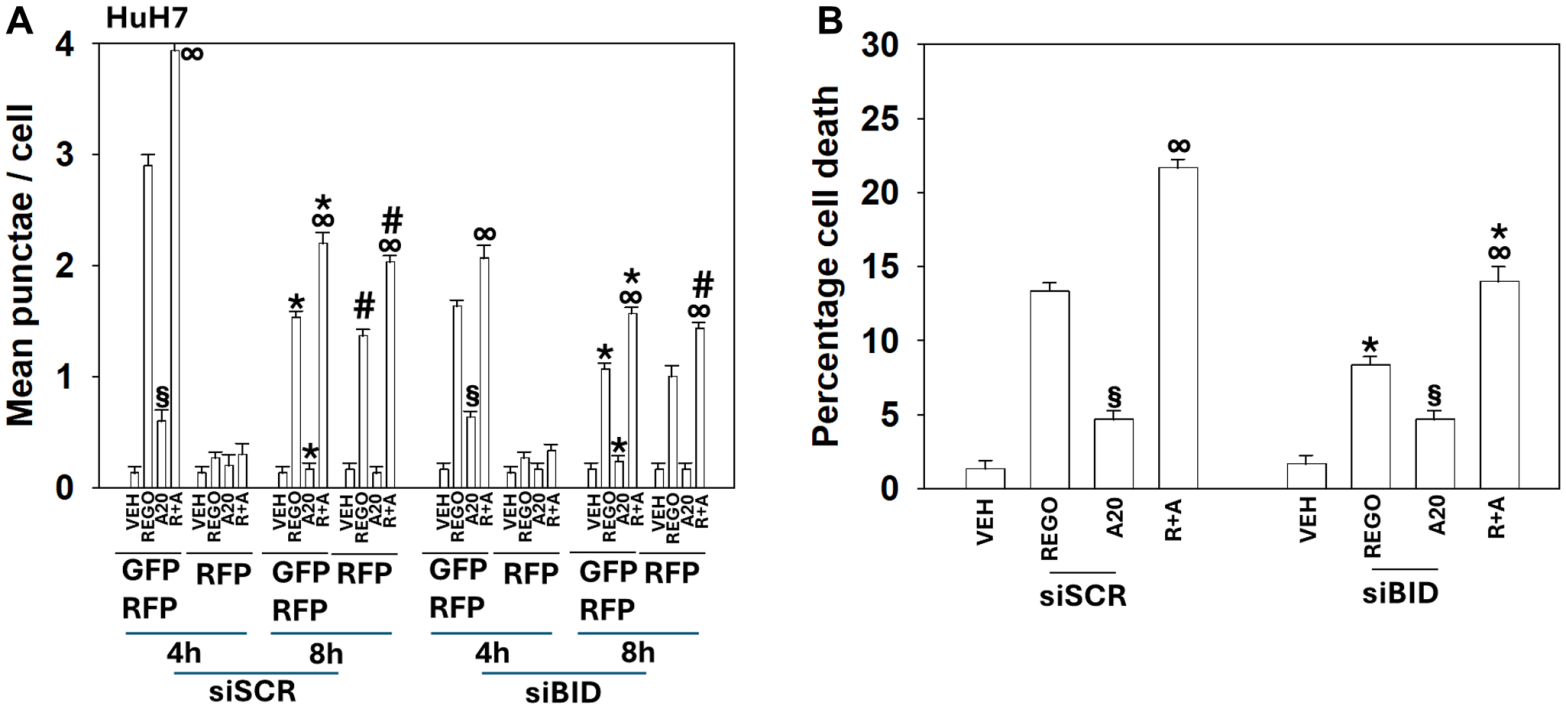
In HuH7 hepatoma cells knock down of BID reduces aramchol-induced autophagic flux and tumor cell death. (**A**) Cells were transfected to express LC3-GFP-RFP and transfected to knock down BID expression. Twenty-four hours later, cells were treated with drugs for 4 h and 8 h. The mean numbers of intense (GFP + RFP) and (RFP alone) vesicles from 100 randomly selected cells were determined. (*n* = 3 ± SD). ^∞^ *p* < 0.05 greater than regorafenib alone value; ^*^ *p* < 0.05 less than corresponding value at the 4 h time point; ^#^ *p* < 0.05 greater than corresponding values at the 4 h time point; ^§^ *p* > 0.05 comparing vesicle formation in siSCR and siBID cells. (**B**) Cells were transfected with an siRNA to knock down expression of BID. Cells were treated with drugs for 24 h. The percentage viability was determined using trypan blue exclusion assays. ^∞^ *p* < 0.05 greater than regorafenib alone value; ^*^ *p* < 0.05 less than corresponding value in siSCR cells; ^§^ *p* > 0.05 comparing vesicle formation in siSCR and siBID cells.

We next determined whether drug-induced macroautophagy was dependent on the actions of specific signaling proteins. Knock down of ATM or AMPKα, proteins activated in both T300/T300 and A300/A300 cells to a similar extent, significantly reduced the formation of autophagosomes and subsequently autolysosomes (Figure 6). The ability of aramchol to further enhance autophagosome/autolysosome formation was still present in cells with either ATM or AMPKα knocked down. Prior regorafenib studies had linked drug toxicity not only to macroautophagy, but also to death receptor signaling [14]. Knock down of the death receptor CD95 or the docking protein FADD significantly reduced the drug-induced formation of autophagosomes and subsequently autolysosomes. Knock down of BID, which is downstream of CD95 and FADD, reduced the numbers of autophagosomes induced by regorafenib as a single agent whereas it had no effect on autophagosome formation caused by aramchol; regorafenib and aramchol, regardless of BID expression, interacted to increase autophagosome levels and to increase autolysosome levels (Figures 7 and 8).

Knock down of either Beclin1 or ATG5, essential proteins for autophagosome formation, significantly reduced autophagosome formation below that observed for any of the other siRNA knock downs and knock down of either Beclin1 or ATG5 almost abolished the formation of autolysosomes. Based on our autophagy data we determined whether the role of BID in tumor cell killing was due to canonical signaling from death receptor CD95/FADD though caspase 8 to cleave BID, or through a different mechanism. Knock down of BID reduced regorafenib lethality as a single agent, but not that of aramchol; aramchol and regorafenib, regardless of BID expression, interacted to cause more tumor cell death (Figures 7 and 8).

**Figure 9:**
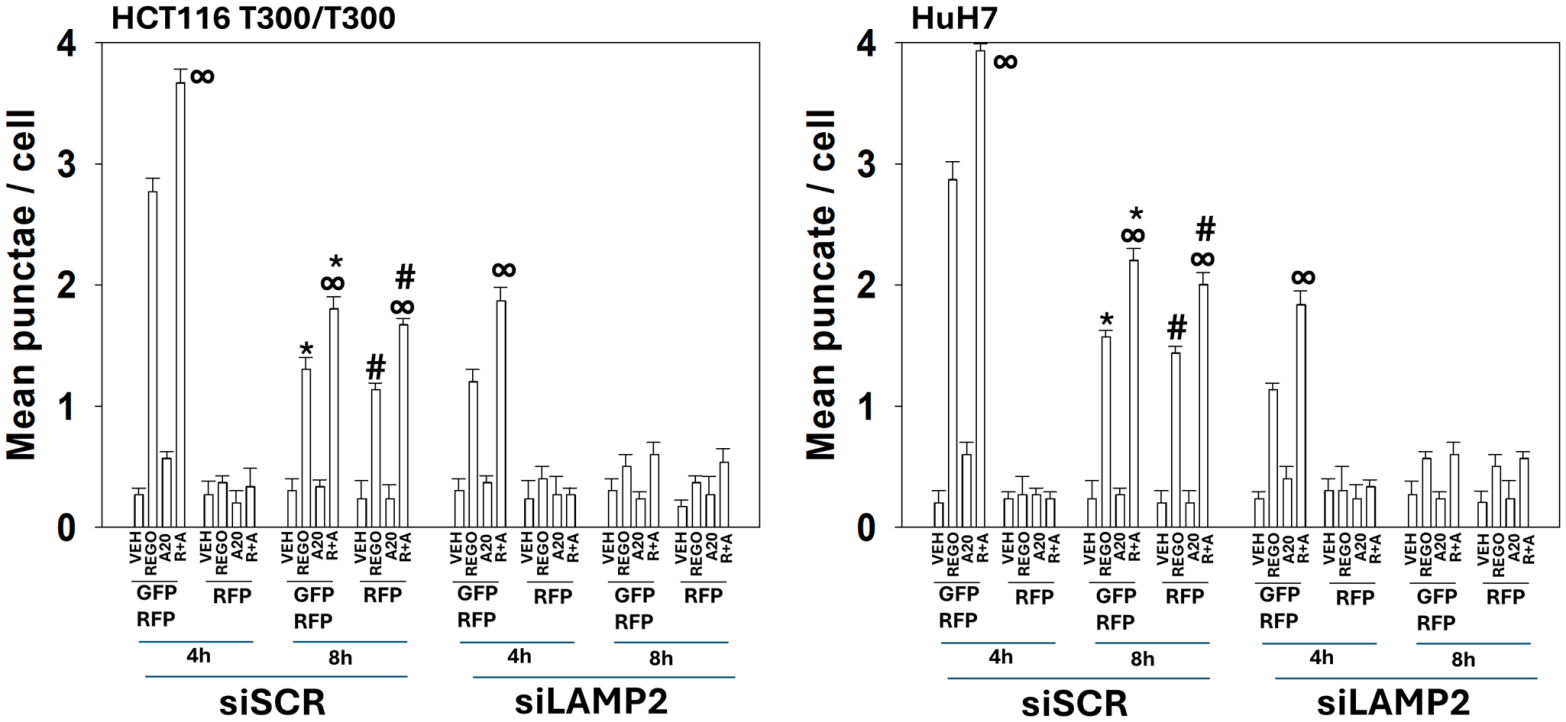
Autolysosome formation requires LAMP2. T300/T300 cells and HuH7 cells were transfected to knock down the expression of LAMP2. After 24 h, cells were treated with drugs for 4 h and 8 h. At each time point, the mean numbers of intense (GFP + RFP) and (RFP alone) vesicles from 100 randomly selected cells were determined. (*n* = 3 ± SD). ^∞^ *p* < 0.05 greater than regorafenib alone value; ^*^ *p* < 0.05 less than corresponding value at the 4 h time point; ^§^ *p* < 0.05 less than corresponding values in T300 cells; ^#^ *p* < 0.05 greater than corresponding values at the 4 h time point.

The protein LAMP2 plays a key role in the formation and maturation of autolysosomes, i.e., it regulates autophagic flux [27]. Knock down of LAMP2 reduced the amount of initial autophagosome formation and abolished the formation of autolysosomes after drug exposure (Figure 9). Knock down of LAMP2 did not lower aramchol-induced killing as a single agent however it did lower the lethality of regorafenib and abolished the toxic interaction between aramchol and regorafenib.

**Figure 10:**
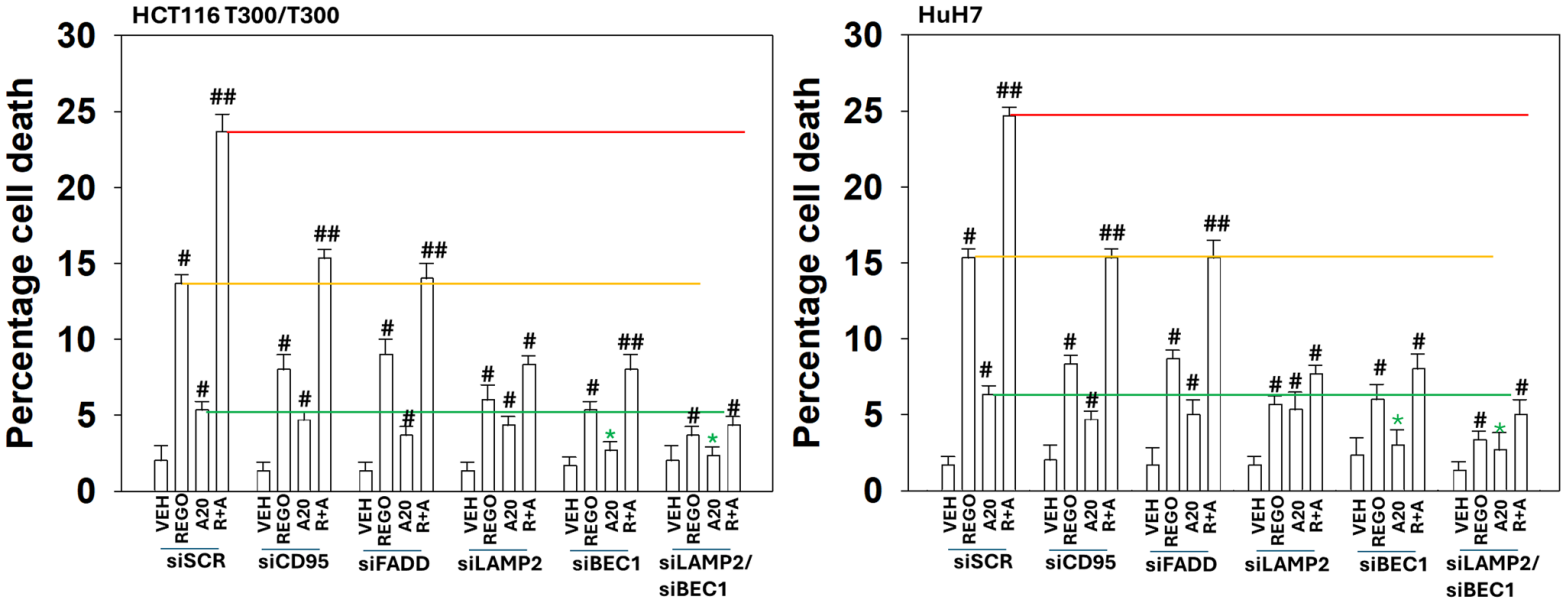
Enhanced LAMP2-dependent autolysosome formation is essential for the lethal interaction between aramchol and regorafenib. HCT116 ATG16L1 T300/T300 and HuH7 adult hepatoma cells were transfected to knock down the expression of CD95, FADD, LAMP2 or Beclin1. Cells were treated with drugs for 24 h. The percentage viability was determined using trypan blue exclusion assays. ^#^ *p* < 0.05 greater than vehicle control; ^##^ *p* < 0.05 greater than regorafenib alone value; ^¶^ *p* < 0.05 less than corresponding value in HCT116 ATG16L1 A300/A300 cells; ^*^ *p* < 0.05 less than corresponding value in siSCR cells.

We then performed studies to link autophagy-regulatory proteins to their roles in tumor cell killing caused by regorafenib and aramchol. The reduction in autophagosome formation observed when CD95 or FADD was knocked down was mirrored in the ability of CD95 or FADD to kill tumor cells (Figure 10). Knock down of Beclin1 almost abolished the formation of both autophagosomes and autolysosomes and it reduced the killing caused by aramchol and regorafenib as single agents and when combined. However, Beclin1 knock down did not abolish the toxic interaction between aramchol and regorafenib. When LAMP2 and Beclin1 were simultaneously knocked down, autophagy and killing were both profoundly reduced. Note that Beclin1, but not LAMP2 knock down, abolished aramchol single agent lethality. Thus, aramchol kills by forming toxic autophagosomes whereas it interacts with regorafenib to kill by enhancing the autophagic flux capability of regorafenib and facilitating greater autolysosome formation.

The weaker biologic responses after drug exposure in A300/A300 cells led us to examine the expression and biological actions of SCD1, the stated target of aramchol, in T300/T300 and A300/A300 cells [28]. Basal levels of SCD1 in T300/T300 and A300/A300 cells were not significantly different (not shown). Treatment of T300/T300 cells, but not A300/A300 cells, with aramchol significantly reduced SCD1 expression (Table 2). As a single agent regorafenib did not alter SCD1 expression, but it combined with aramchol to further reduce SCD1 expression. Knock down of Beclin1 or ATG5 prevented aramchol as a single agent and when combined with regorafenib from reducing SCD1 expression (Table 3). Thus, despite regorafenib causing greater levels of autophagosome formation than aramchol, SCD1 could only be degraded by macroautophagy when it had first been “destabilized” by aramchol. Based on this data, with SCD1 being catalytically inhibited and also downregulated by aramchol, we performed preliminary lipidomic analyses.

**Table 2:** Aramchol reduces the expression of SCD1, which is enhanced by regorafenib

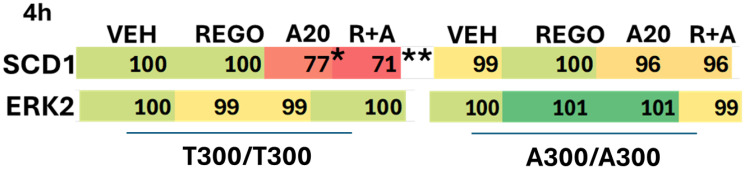

HCT116 ATG16L1 T300/T300 cells and HCT116 ATG16L1 A300/A300 cells were treated with vehicle control, aramchol (20 μM), regorafenib (0.5 μM) or the drugs combined for 4 h. The percentage alteration in expression of SCD1 caused by the drugs was determined from three independent replicates (±SD). Values with a *p* < 0.05 were considered significant. ^*^ *p* < 0.05 less than vehicle control; ^**^ *p* < 0.05 less than aramchol alone value.

**Table 3:** Aramchol reduces the expression of SCD1 via macroautophagy

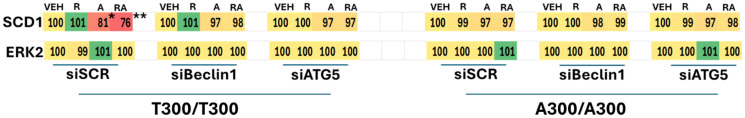

HCT116 ATG16L1 T300/T300 cells and HCT116 ATG16L1 A300/A300 cells were transfected with a scrambled siRNA (siSCR) or with siRNA molecules to knock down the expression of Beclin1 or ATG5. The percentage alteration in expression of SCD1 caused by the drugs was determined from three independent replicates (±SD). Values with a *p* < 0.05 were considered significant. ^*^ *p* < 0.05 less than vehicle control; ^**^ *p* < 0.05 less than aramchol alone value.

As aramchol is an SCD1 inhibitor it would be a priori predicted that we would in parallel observe alterations in the levels of various lipid moieties. HCT116 and HuH7 cells were treated with aramchol and regorafenib and isolated at the time of peak autophagosome formation, 4 hours. Mass spectrometry analyses of the lipids under each treatment condition were determined. In both cell lines, aramchol as a single agent increased the levels of ceramide-1-phosphate and of triglycerides (Supplementary Figure 3). Heat map comparative analyses also demonstrated increased levels of ceramide-1-phosphate and triglycerides, as well as reduced levels of some phosphatidyl choline species, whilst other species were significantly increased (Supplementary Figures 4 and 5).

**Figure 11:**
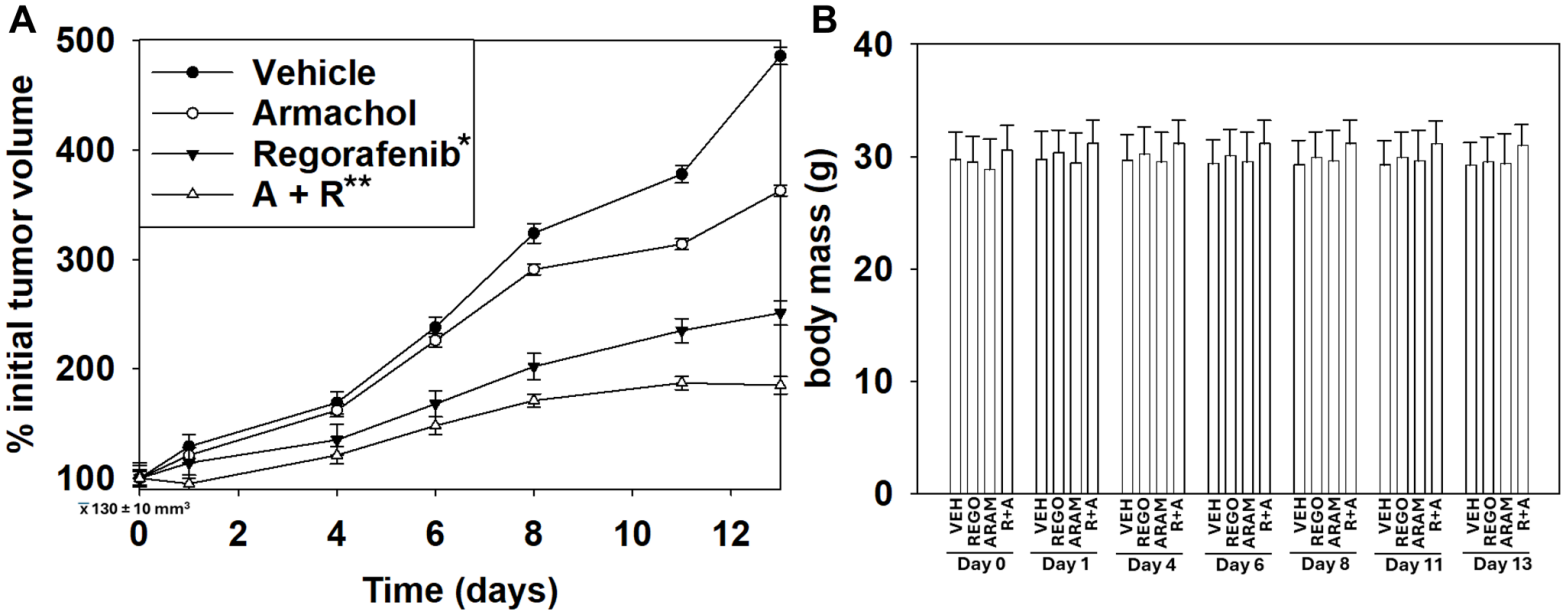
Aramchol and regorafenib interact to suppress tumor growth *in vivo*. (**A**) Studies were performed *per* USDA regulations under approved VCU IACUC protocol AD20008. HuH7 adult hepatoma cells (2 × 10^6^) were injected into the left rear and right rear flanks of male NRG mice. Over two weeks, tumors formed: mean volume 130 ± 10 mm^3^. Animals were treated with vehicle control or with drugs IP daily; aramchol (50 mg/kg), regorafenib (10 mg/kg) or the drugs in combination. Tumor volumes were calculated on the indicated days using the formula (length – longest diameter) × (width)^2^/2 (*n* = 20 ± SD) ^*^ *p* < 0.05 less than vehicle control; ^**^ *p* < 0.05 less than aramchol as a single agent. (**B**) Mouse body mass was determined daily and is presented for those days when tumor volumes were, in parallel, being determined (*n* = 10 ± SD). There was no significant alteration in mouse body mass over the time course, *p* > 0.05.

Finally, we determined whether aramchol and regorafenib safely interacted in a mouse tumor model. Regorafenib and aramchol interacted to significantly reduce the growth of HuH7 hepatoma tumors in a flank model (Figure 11A). It was notable that as a single agent, the anti-tumor actions of aramchol only became evident approximately a week after the start of dosing. During the study, the drugs alone or combined did not significantly alter the body mass of the mice compared to control (Figure 11B).

## DISCUSSION

The present investigations were performed as a pre-clinical developmental cancer therapeutics assessment of the most efficacious combination of the SCD1 inhibitor aramchol combined with a multi-kinase inhibitor in GI tumor cells. Aramchol is a non-toxic synthetic bile acid being developed for the treatment of liver disease. Comparing sorafenib, regorafenib and lenvatinib, we discovered that the aramchol regorafenib combination most effectively killed GI tumor cells.

We discovered that regorafenib and to a slightly lesser extent aramchol activated ATM and the AMPK, and they interacted to cause more activation of both kinases [5]. Regorafenib and aramchol also interacted to cause greater inactivation of mTORC1 and mTORC2. Regorafenib as a single agent significantly increased ATG13 S318 phosphorylation whereas aramchol did not, and no significant interaction was observed to increase S318 phosphorylation when the drugs were combined. As expected, based on it elevating ATG13 S318 levels, regorafenib as a single agent strongly increased autophagosome formation. More surprisingly was that although there was no significant increase in ATG13 S318 phosphorylation caused by aramchol, it could nevertheless also more modestly increase autophagosome levels. And again, despite not observing a drug interaction to further increase ATG13 S318 phosphorylation, aramchol and regorafenib interacted to cause greater amounts of autophagosome formation. This argues that aramchol acts to enhance autophagy through unknown mechanisms that are different to those of regorafenib.

The key molecular mechanisms by which aramchol and regorafenib killed GI tumor cells were defined. Aramchol as a single agent increased autophagosome formation but did not enhance autophagic flux, though it did further enhance the formation of autophagosomes, and autolysosomes that were caused by regorafenib. The interaction between aramchol and regorafenib, causing more autophagic flux and autolysosome formation, was required for the enhanced killing of tumor cells by the drug combination (Supplementary Figure 5).

We observed that knock down of the death receptor CD95 or its docking protein FADD, reduced the amount of drug-induced autophagosome and autolysosome formation. Knock down of the toxic BH3 domain protein BID, which is downstream of CD95, FADD and pro-caspase 8, significantly reduced the ability of regorafenib alone or in combination with aramchol to cause autophagosome and autolysosome formation and to kill tumor cells. BID knock down did not alter autophagosome formation or cell killing caused by aramchol as a single agent. This is further evidence that the mechanisms of action of aramchol are different to those of regorafenib. How aramchol can increase autophagosome formation in the absence of enhanced ATG13 S318 still need to be resolved.

Although the genomes of all members of *Homo sapiens sapiens* are near-identical, modest genetic variations between individuals at a specific location or between different populations located in geographically distant parts of the planet can confound simplistic a priori assumptions of how persons may respond to novel therapies in the clinic, such as the isoform dimorphism of ATG16L1. Our interest in the macroautophagy-regulatory protein ATG16L1 was sparked by the finding that alanine homozygosity at residue 300 of ATG16L1 (A300/A300) is more predominant in whites and threonine homozygosity at the same residue (T300/T300) more predominant in African Americans [29]. T300/T300 homozygosity was shown to play a key role in why African Americans have less incidence of Crohn’s Disease compared to whites. Furthermore, compared to non-Hispanic Whites, African Americans are 20% more likely to get colorectal cancer and 40% more likely to die from the disease [30]. In colorectal cancer, the A300/A300 genotype is associated with reduced metastasis and increased overall survival [30, 31]. Hence, the role of ATG16L1 isoform status in the regulation of tumor cell survival after a therapeutic intervention may likely play a key role patient survival.

Using HCT116 colon cancer cells homozygous for the T300 isoform or the A300 isoform of ATG16L1, we determined, comparing the signaling pathways activated by the drugs, that greater alterations in protein phosphorylation and protein expression were observed in T300/T300 cells compared to A300/A300 cells. The formation of autophagosomes, autophagic flux, and the formation of autolysosomes were greater in T300/T300 cells compared to A300/A300 cells. The ability of regorafenib, aramchol and in combination to kill T300/T300 cells was significantly higher than in A300/A300 cells; cells lacking ATG16L1 expression were killed less effectively than A300/A300 cells. The molecular mechanisms explaining why A300/A300 cells did not respond in their signaling, autophagy and cell death parameters as efficaciously to regorafenib and aramchol compared to T300/T300 cells remain to be determined.

In T300/T300 cells and to a lesser extent in A300/A300 cells regorafenib increased endoplasmic reticulum stress signaling as judged by elevated phosphorylation of PERK and eIF2α, and increased expression of serine/threonine protein phosphatase 1 and GRP78. Unexpectedly, however, we did not observe increased expression of the intermediary signaling molecules downstream of eIF2α, ATF4 and CHOP. The reasons why eIF2α phosphorylation in this system did not regulate ATF4 and CHOP are unclear and additional studies, e.g., RNA-seq, will be required to identify alternative downstream eIF2α effectors.

It has been shown by several groups that prolonged exposure of SCD1 to catalytic inhibitors of the enzyme ultimately causes the degradation of the protein. Our original hypothesis was that both regorafenib and aramchol would downregulate SCD1 and interact to cause more degradation. Instead, whilst aramchol did reduce SCD1 levels, regorafenib as a single agent had no effect. Regorafenib causes greater amounts of autophagosome formation, flux and autolysosome formation whereas aramchol weakly stimulates autophagosome formation with no flux. From this we concluded that SCD1 could only be degraded by autophagy when it had first been destabilized by aramchol.

Aramchol is proposed to specifically inhibit SCD1, however, based on our experiences with other therapeutic agents, it is very probable that this drug also has other unknown targets. As such, for our initial lipidomic studies, we took an agnostic view to data acquisition and examined all lipid moieties detected by the mass spectrometer. Aramchol as a single agent increased the levels of ceramide-1-phosphate and of triglycerides. A heat map with comparative analyses additionally demonstrated that the levels of phosphatidyl choline species with different chain lengths were either enhanced or reduced. Similar findings were made for the levels of different ceramides. The primary mode of generating ceramide-1-phosphate has been proposed to be via ceramide kinase, though as phosphatases are inherently an order of magnitude more catalytically active than kinases, a small reduction in the dephosphorylation of ceramide-1-phosphate would also result in a substantial increase in ceramide-1-phosphate levels.

Generally, the biology of ceramide-1-phosphate in oncology has previously linked this phospholipid to the proliferation, invasion and therapeutic resistance of tumor cells, which is the opposite of the biology we observed treating cells with aramchol, and more so aramchol plus regorafenib [32–34]. One possible explanation of this discrepancy may be based on the location of the ceramide-1-phosphate that is being generated [35–39]. Ceramide-1-phosphate transfer protein (CPTP) is a glycolipid transfer protein which specifically binds ceramide-1-phosphate rather than other phospho-sphingolipids, e.g., sphingosine-1-phosphate. Ceramide-1-phosphate is synthesized in the Golgi by ceramide kinase and CPTP translocates the ceramide-1-phosphate to other membranes, including those of autophagosomes, regulating autophagy. Accumulation of ceramide-1-phosphate in the Golgi can occur when CPTP expression is reduced, and which ultimately results in elevated levels of pro-inflammatory lipids such as eicosanoids. Eicosanoids can act to enhance autophagy [33, 39]. Further analyses using targeting specifically labeled lipid moieties will be required to fully understand the roles of bioactive lipids in cancer cells treated with aramchol and regorafenib [34–36].

Aramchol is primarily distributed in the liver and adipose tissues. We initially performed mouse model studies using a flank model to determine whether the residual circulating aramchol in the plasma could interact with regorafenib to suppress tumor growth. As aramchol tends to concentrate in the liver, flank studies are sub-optimal to a liver-localized tumor orthotopic model, but are less invasive and more straightforward to perform. Aramchol interacted with regorafenib to suppress tumor growth and did so without causing mice to significantly lose body mass. As a single agent, regorafenib immediately began to suppress tumor growth whereas it took over seven days before aramchol as a single agent exhibited any anti-tumor effects. Based on the data in this manuscript, a protocol is being written for the drug combination of aramchol and regorafenib to be translated into the clinic.

## MATERIALS AND METHODS

### Materials

The hepatoma cell lines HEP3B and HuH7 cells were purchased from Biohippo Inc. (Gaithersburg, MD, USA). HCT116 wild type T300/T300 and isogenic HCT116 A300/A300 cells were kindly provided by Dr. David Boone. Sorafenib, regorafenib and lenvatinib were purchased from Selleckchem (Houston, TX, USA). Aramchol was provided by Galmed Pharmaceuticals (Tel Aviv, Israel). Trypsin-EDTA, DMEM, RPMI, penicillin-streptomycin were purchased from GIBCOBRL (GIBCOBRL Life Technologies, Grand Island, NY, USA). The LC3-GFP-RFP plasmid was obtained from Addgene (Watertown, MA, USA; #117413). Antibodies were purchased from Cell Signaling Technology (Danvers, MA, USA); Abgent (San Diego, CA, USA); Novus Biologicals (Centennial, CO, USA); Abcam (Cambridge, UK); and Santa Cruz Biotechnology (Dallas, TX, USA). No human studies were performed as a component of this manuscript.

### Methods

All bench-side Methods used in this manuscript have been previously performed and described in the peer-reviewed references [18–21]. Briefly, cells where indicated were transfected with a scrambled siRNA control (siSCR) or with validated siRNA molecules to knock down the expression of the indicated proteins. After 24 h, cells were treated with vehicle control, regorafenib (0.5 μM), aramchol (20 μM)] or the drugs combined for 4 h. Cells were fixed in place, permeabilized and subjected to in-cell immunostaining for the indicated proteins/phospho-proteins. Cells were imaged using an Odyssey infrared imager. The percentage alteration in expression/phosphorylation caused by the drugs was determined from three independent replicates (±SD). Values with a *p* < 0.05 were considered significant.

### Detection of cell death by trypan blue assay

Cells where indicated were transfected with a scrambled siRNA control (siSCR) or with validated siRNA molecules to knock down the expression of the indicated proteins. After 24 h, cells were treated with vehicle control, regorafenib (0.5 μM), aramchol (20 μM) or the drugs combined for 24 h. The number of dead cells was counted and expressed as a percentage of the total number of cells counted (*n* = 3 ± SD) [14–16, 18, 20, 21, 24].

### Transfection of cells with siRNA/plasmids

Cells were plated and 24 h after plating, transfected. A plasmid to express LC3-GFP-RFP was used throughout the study (Addgene, Waltham, MA, USA). Control studies to define the percentage of protein knock down caused by siRNA molecules are presented in Supplementary Figure 6 [14–16, 18, 20, 21, 24].

### Assessments of autophagosome and autolysosome levels

Cells were transfected with a plasmid to express LC3-GFP-RFP. Twenty-four hours after transfection, cells were treated with vehicle control, regorafenib (0.5 μM), aramchol (20 μM) or the drugs combined for 4 h and for 8 h. Cells were imaged at 60X magnification and the mean number of (GFP+RFP+) and (RFP+) punctae per cell determined in living cells from >100 randomly selected cells per condition (*n* = 3 ± SD) [14–16, 18, 20, 21, 24].

### Untargeted lipidomic profiles

These were performed as described in references [22, 40].

### Animal studies

Studies were performed per USDA regulations under approved VCU IACUC protocol AD20008. HuH7 adult hepatoma cells (2 × 10^6^) were injected into the left rear and right rear flanks of male NRG mice. Over two weeks, tumors formed: mean volume 130 ± 10 mm^3^. Animals were treated with vehicle control or with drugs IP daily; aramchol (50 mg/kg), regorafenib (10 mg/kg) or the drugs in combination. Tumor volumes were calculated on the indicated days using the formula (length – longest diameter) × (width)^2^/2. Mouse body mass was determined daily and is presented for those days when tumor volumes were in parallel being determined.

### Data analysis

Comparison of the effects of various treatments was using one-way ANOVA for normalcy followed by a two tailed Student’s *t*-test with multiple comparisons. Differences with a *p*-value of < 0.05 were considered statistically significant. Experiments are the means of multiple individual data points per experiment from 3 independent experiments (±SD).

## SUPPLEMENTARY MATERIALS



## Abbreviations

ERK: extracellular regulated kinase
PI3K: phosphatidyl inositol 3 kinase
ER: endoplasmic reticulum
AMPK: AMP-dependent protein kinase
mTOR: mammalian target of rapamycin
JAK: Janus Kinase
STAT: Signal Transducers and Activators of Transcription
MAPK: mitogen activated protein kinase
PTEN: phosphatase and tensin homologue on chromosome ten
si: small interfering
SCR: scrambled
VEH: vehicle
REG: regorafenib
ARA: aramchol
