# Supplementary material for: The SCD1 inhibitor aramchol interacts with regorafenib to kill GI tumor cells in vitro and in vivo
Source: Oncotarget. 2025 Aug 19;16:662–78. doi: 10.18632/oncotarget.28762 (PMC12581408; doi:10.18632/oncotarget.28762)
Supplement: Supplementary file 1 [file oncotarget-16-28762-s001.pdf]

The SCD1 inhibitor aramchol interacts with regorafenib to kill GI tumor cells *in vitro* and *in vivo*

SUPPLEMENTARY MATERIALS

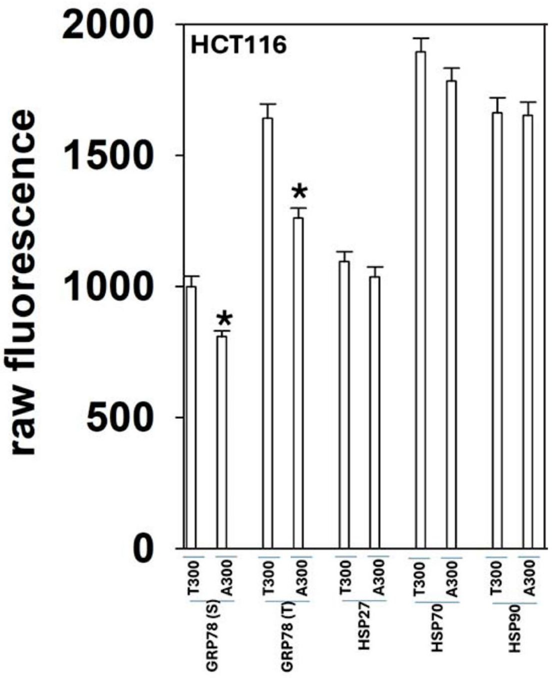

Supplementary Figure 1: Basal GRP78 expression is lower in HCT116 ATG16L1 A300/A300 cells. HCT116 cells, T300/T300 and A300/A300, were fixed in place and in-cell immunostaining for the indicated proteins was performed. ($n = 3 \pm$ SD) $^{*}p < 0.05$ less than corresponding value in T300/T300 cells.

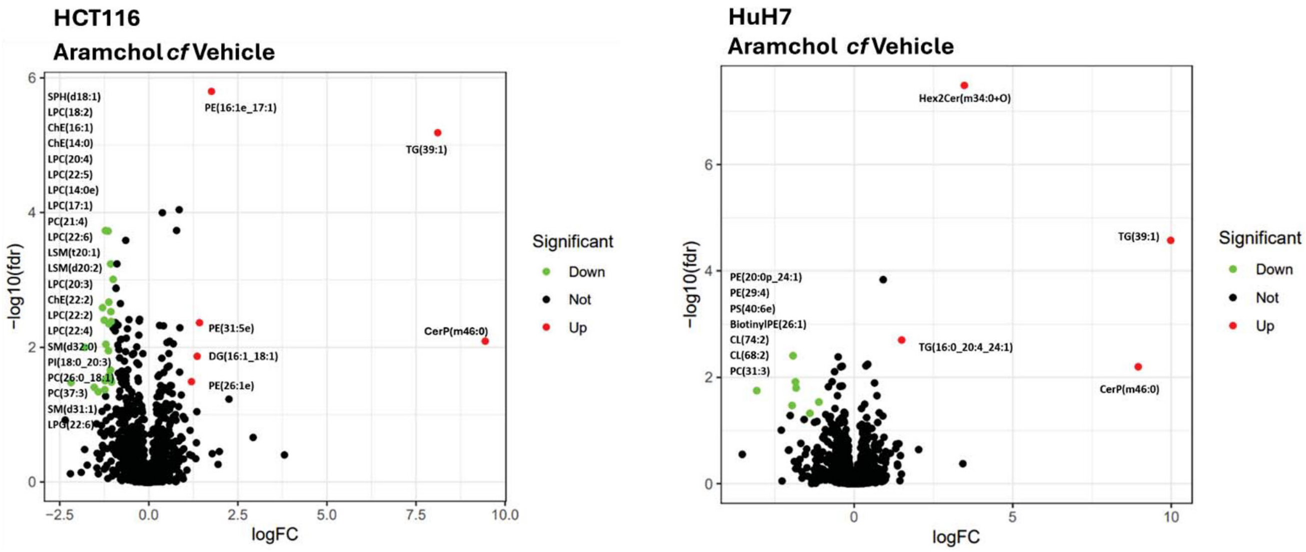

Supplementary Figure 2: Lipidomic volcano plot analyses of HCT116 and HuH7 cells treated with aramchol and regorafenib. HCT116 ATG16L1 T300/T300 cells and HuH7 cells were treated with vehicle control, regorafenib (2 μM), aramchol (20 μM) or the drugs combined for 4 h. Cells were processed for lipidomic analyses as described in the Methods section ($n = 3$, ± SD). All colored values are $p < 0.05$ change compared to vehicle control value.

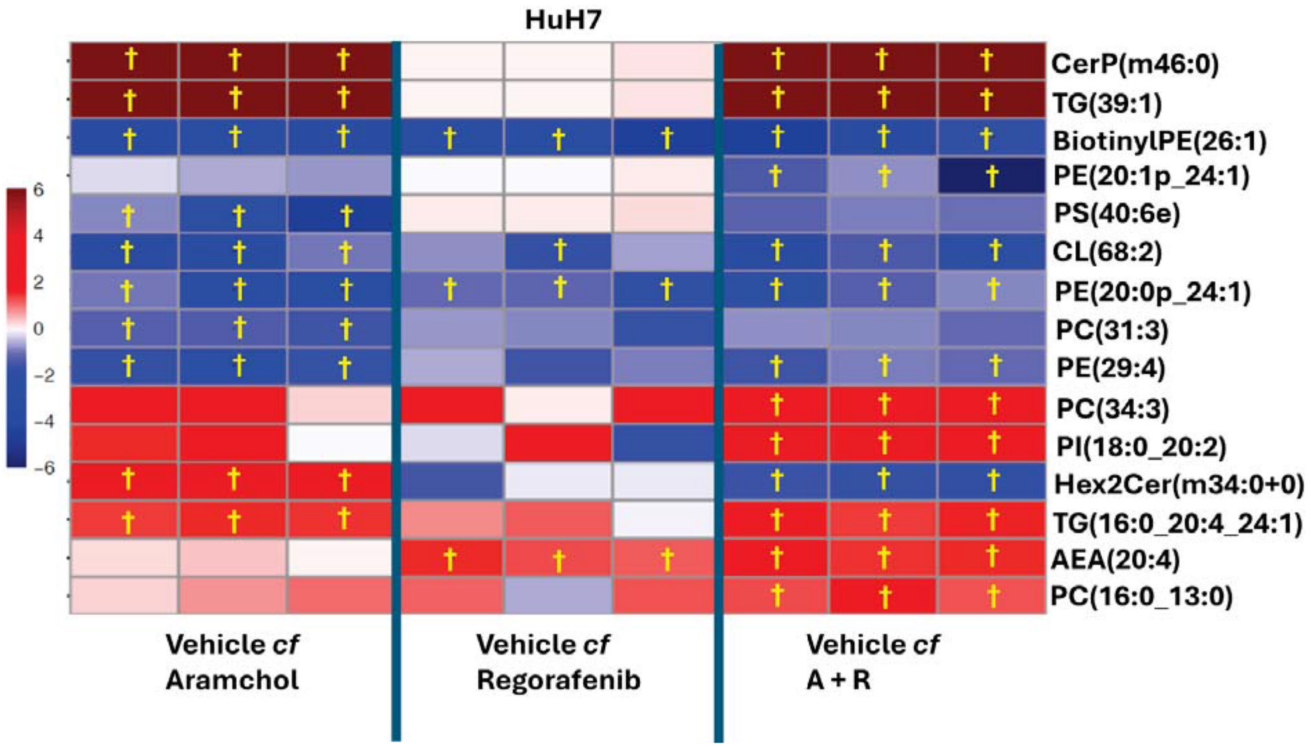

Supplementary Figure 3: Representative heatmap lipidomic analyses of HCT116 cells treated with aramchol and regorafenib. HCT116 ATG16L1 T300/T300 cells were treated with vehicle control, regorafenib (2 μM), aramchol (20 μM) or the drugs combined for 4 h. Cells were processed for lipidomic analyses as described in the Methods section ($n = 3$, ± SD) $^{\dagger}p < 0.05$ change compared to vehicle control value.

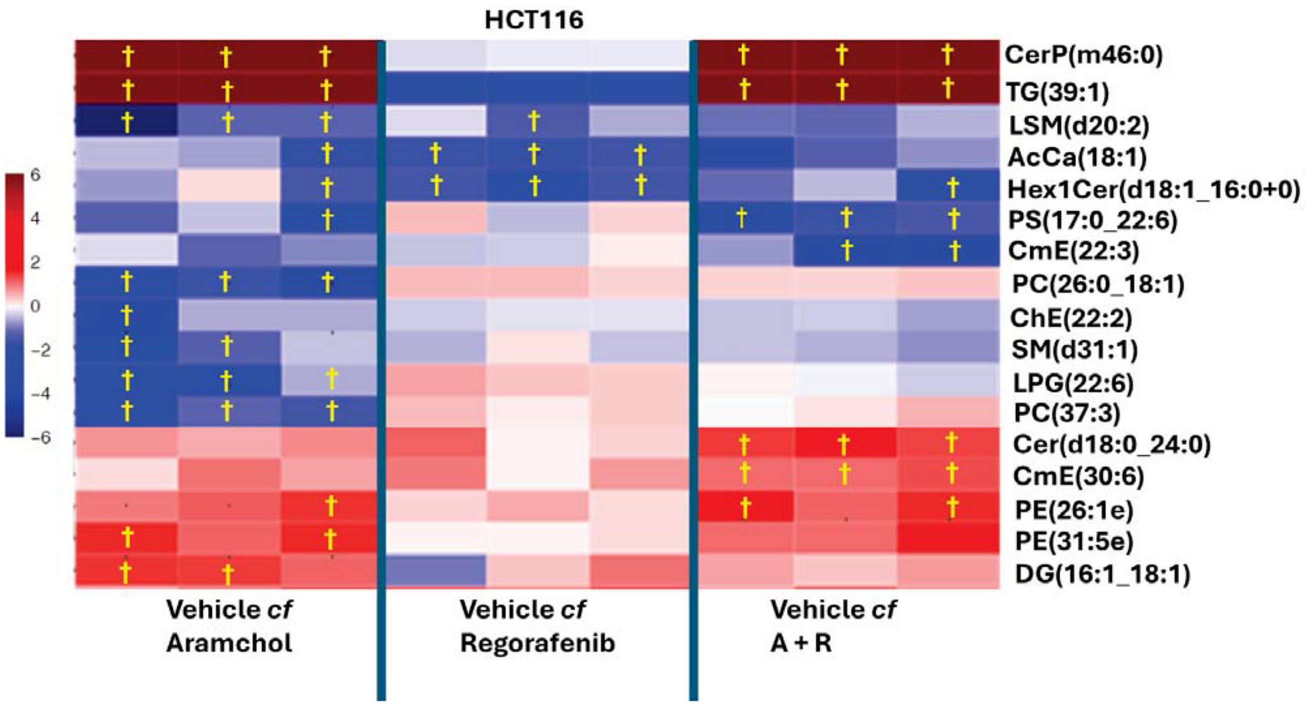

Supplementary Figure 4: Representative heatmap lipidomic analyses of HuH7 cells treated with aramchol and regorafenib. HuH7 cells were treated with vehicle control, regorafenib (2 μM), aramchol (20 μM) or the drugs combined for 4 h. Cells were processed for lipidomic analyses as described in the Methods section ($n = 3$, ±SD) $^{\dagger}p < 0.05$ change compared to vehicle control value.

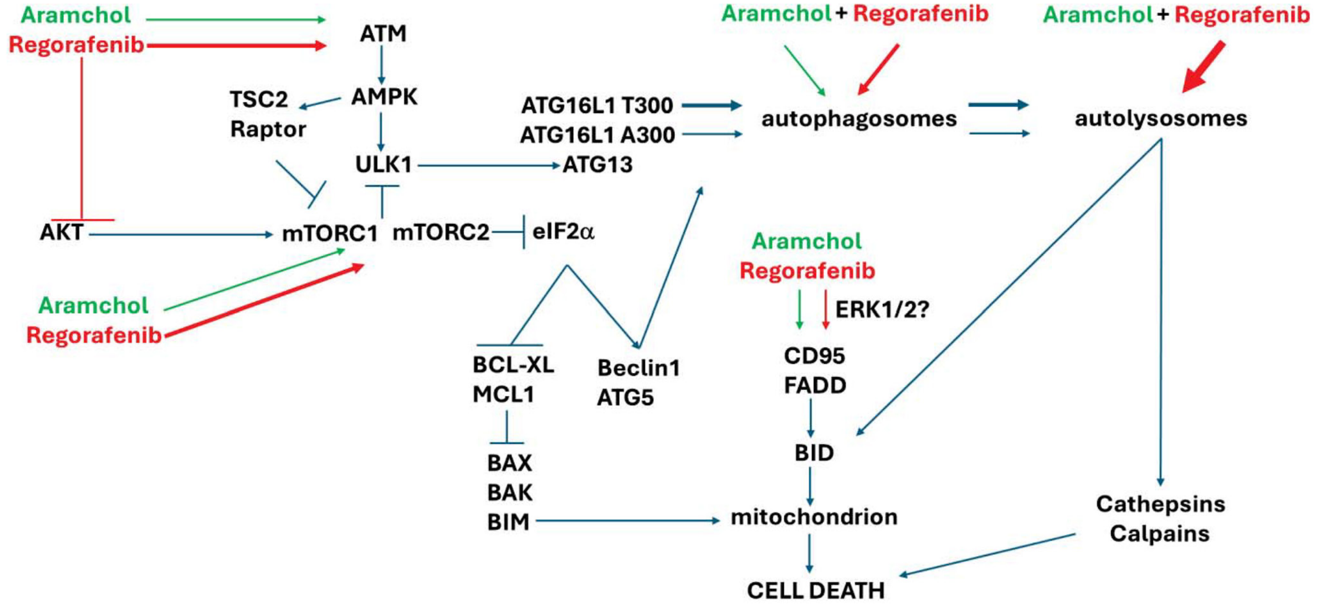

Supplementary Figure 5: A schematic of how aramchol and regorafenib may interact to kill tumor cells. In cells homozygous for the autophagy-regulatory protein ATG16L1 T300, aramchol and regorafenib interacted to activate ATM and the AMPK and to inactivate mTORC1 and mTORC2. As a single agent, regorafenib inactivated eIF2α leading to reduced expression of MCL1 and BCL-XL. In cells expressing the ATG16L1 A300 isoform the drug-induced dephosphorylation of mTORC1 S2448 and mTORC2 S2481 and the increased phosphorylation of eIF2α S51 were significantly lower than in T300 cells. In cells expressing ATG16L1 T300, but not A300, regorafenib and/or the drug combination inactivated AKT and ERK1/2, this may be responsible for CD95 activation. Regorafenib and aramchol interacted to cause formation of autophagosomes which was significantly greater in cells expressing ATG16L1 T300. Aramchol as a single agent did not stimulate autophagic flux but further enhanced both flux and autolysosome formation caused by regorafenib. Knock down of Beclin1 reduced the lethality of regorafenib and aramchol as single agents and when combined whereas knock down of LAMP2 or BID did not reduce killing caused by aramchol as a single agent but did reduce the lethality of regorafenib alone and regorafenib plus aramchol.

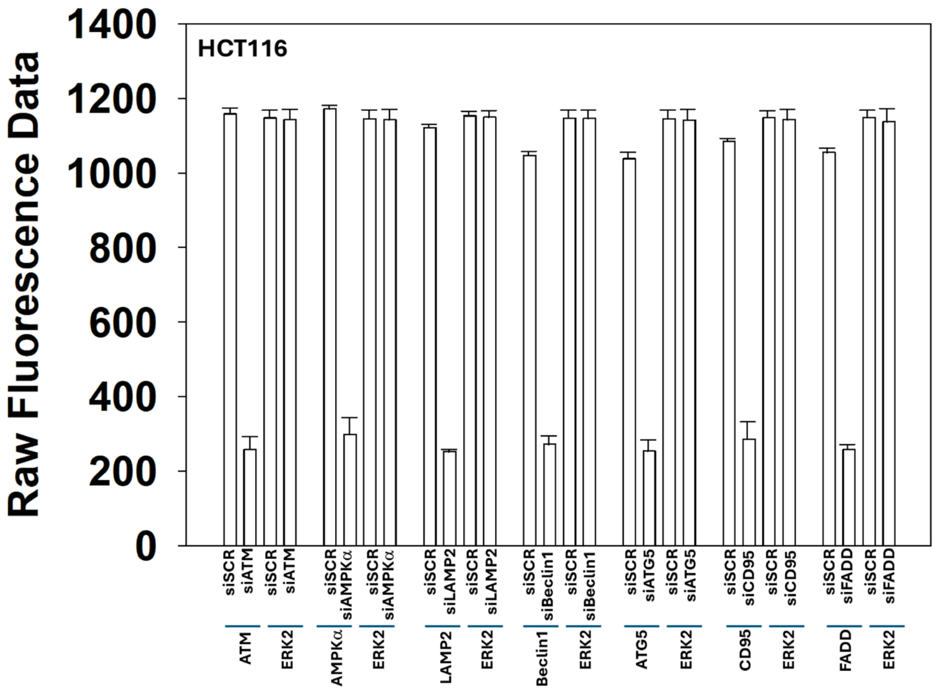

Supplementary Figure 6: Control data demonstrating siRNA knock down of proteins. HCT116 ATG16L1 T300/T300 cells were transfected with a scrambled control siRNA (siSCR) or with an siRNA to knock down each specific protein, as indicated. After 24 h, cells were fixed in place an in-cell immunostaining performed to detect the protein levels of each protein, as indicated. Parallel staining for ERK2 was performed as a loading control (*n* = 3 independent assessments, ±SD).

Supplementary Table 1: Aramchol and regorafenib interact to activate ATM, AMPK and inactivate mTORC1 in HCT116 cells

	T300/T300				A300/A300			
4h	VEH	REG	ARA	R+A	VEH	REG	ARA	R+A
ATM	100	99	100	100	100	99	100	100
P-ATM S1981	100	127#	112#	134##	100	121#	108	131##
AMPKα	100	100	99	100	100	100	99	100
P-AMPKα T172	100	125#	116#	133##	100	118#	111	128##
mTOR	100	100	101	101	100	100	101	100
P-mTORC1 S2448	100	76*	92	66**	100	84*†	96	84*†
P-mTORC2 S2481	100	80*	91	69**	100	90*†	100	85*†
ERK2	100	100	100	100	100	100	100	99
ULK1	100	100	100	100	100	100	99	99
P-ULK1 S757	100	83*	95	77*	100	87*	99	84*†
P-ULK1 S317	100	127#	103	131#	100	112#†	102	116#†
eIF2α	100	100	101	101	100	99	99	100
P-eIF2α S51	100	142#	112	147#	100	118#†	104	121#†
PERK	100	101	100	100	100	99	99	99
P-PERK T980	100	133#	109	136#	100	117#†	105	122#†
ERK2	100	100	101	100	100	101	101	101

	T300/T300				A300/A300			
4h	VEH	REG	ARA	R+A	VEH	REG	ARA	R+A
AKT	100	100	100	105	100	100	100	100
P-AKT T308	100	80*	93	76*	100	97	103	97
STAT3	100	99	99	101	100	100	100	100
P-STAT3 Y705	100	93	98	92	100	98	102	98
STAT5	100	100	101	103	100	101	101	100
P-STAT5 Y694	100	84*	92	82*	100	101	102	98
ERK2	100	100	99	101	100	100	100	99
Beclin1	100	122#	102	126#	100	108	101	110#†
ATG5	100	124#	105	127#	100	106	100	106
ATG13	100	100	100	99	100	101	100	100
P-ATG13 S318	100	119#	108	123#	100	104	103	107
GRP78	100	120#	103	128##	100	116#	107	119#†
CHOP	100	99	97	101	100	106	98	107
PP1	100	113#	99	113#	100	99	100	103
ERK2	100	100	101	100	100	99	100	100

HCT116 T300/T300 cells and HCT116 A300/A300 cells in 96- well plates were treated with vehicle control, aramchol (20 µM), regorafenib (0.5 µM) or the drugs combined for 4 h. Cells were fixed in place, permeabilized and subjected to in-cell immunostaining for the indicated proteins/phospho-proteins. Cells were imaged using an Odyssey infrared imager. ERK2 staining was used as an invariant loading control. The percentage alteration in expression/phosphorylation caused by the drugs was determined from three independent replicates. Values with a $p < 0.05$ were considered significant. $^{\#}p < 0.05$ greater than vehicle control; $^{\#\#}p < 0.05$ greater than regorafenib alone value; $^{*}p < 0.05$ less than vehicle control; $^{**}p < 0.05$ less than regorafenib alone value; $^{\dagger}p < 0.05$ less than corresponding value in HCT116 T300/T300 cells.

Supplementary Table 2: Aramchol and regorafenib interact in HCT116 cells to decrease expression of MCL1 and BCL-XL and to increase the expression of BAX, BAK and BIM

4h	T300/T300 VEH	T300/T300 REG	T300/T300 ARA	T300/T300 R+A	A300/A300 VEH	A300/A300 REG	A300/A300 ARA	A300/A300 R+A
p65 NFκB	100	101	101	100	100	100	100	100
P-p65 NFκB S536	100	85	101	85 *	100	95	100	94 †
c-SRC	100	101	101	101	100	100	100	100
P-c-SRC Y416	100	90	98	86 *	100	95	98	95 †
P-c-SRC Y527	100	105	102	106	100	102	102	103
c-MET	100	100	100	100	100	102	102	102
P-c-MET	100	100	101	97	100	99	99	99
ERK2	100	100	100	100	100	100	100	100
CD95	100	100	99	98	100	100	100	99
FAS-L	100	104	102	106	100	97	101	100
JAK2	100	100	100	101	100	100	100	100
P-JAK2	100	101	97	99	100	100	99	100
c-KIT	100	99	99	100	100	99	99	100
P-c-KIT	100	92	92	91 *	100	100	99	101
ERK2	100	99	100	100	100	101	100	100
PDGFRβ	100	100	100	100	100	100	100	99
P-PDGFRβ	100	90 *	96	85 *	100	106	104	105
HSP70	100	98	101	97	100	100	102	99
HSP90	100	102	101	103	100	99	100	99
PD-L1	100	89 *	98	88 *	100	94	99	93
MHCA	100	113 #	99	115 #	100	99	99	99
ERK2	100	99	100	100	100	100	100	100

4h	T300/T300 VEH	T300/T300 REG	T300/T300 ARA	T300/T300 R+A	A300/A300 VEH	A300/A300 REG	A300/A300 ARA	A300/A300 R+A
BCL-XL	100	84 *	94	79 *	100	90	102	92
MCL1	100	83 *	78 *	71 **	100	93	99	90
BAX	100	115 #	106	117 #	100	105	98	105
BAK	100	110	102	112 #	100	108	106	112 #
BIM	100	104	111 #	119 #	100	104	102	108
ERK2	100	99	100	100	100	101	100	101
LATS1/2	100	100	100	101	100	100	100	100
P-LATS T1097	100	108	105	112 #	100	102	100	101
P-LATS S909	100	104	103	116 #	100	103	105	105
YAP	100	100	99	99	100	100	100	100
P-YAP S109	100	103	102	101	100	103	100	102
P-YAP S127	100	109	108	112 #	100	100	98	99
P-YAP S397	100	111 #	107	112 #	100	100	102	100
TAZ	100	100	100	99	100	100	101	100
P-TAZ S89	100	110	107	114 #	100	102	100	102
ERK2	100	100	100	100	100	100	100	100

HCT116 T300/T300 cells and HCT116 A300/A300 cells in 96-well plates were treated with vehicle control, aramchol (20 µM), regorafenib (0.5 µM) or the drugs combined for 4 h. Cells were fixed in place, permeabilized and subjected to in-cell immunostaining for the indicated proteins/phospho- proteins. Cells were imaged using an Odyssey infrared imager. ERK2 staining was used as an invariant loading control. The percentage alteration in expression/phosphorylation caused by the drugs was determined from three independent replicates. Values with a $p < 0.05$ were considered significant. $^{\#}p < 0.05$ greater than vehicle control; $^{\#\#}p < 0.05$ greater than regorafenib alone value; $^{*}p < 0.05$ less than vehicle control; $^{**}p < 0.05$ less than regorafenib alone value; $^{\dagger}p < 0.05$ less than corresponding value in HCT116 T300/T300 cells.

Supplementary Table 3: Aramchol and regorafenib interact to reduce the phosphorylation of multiple ERBB receptor family proteins

	T300/T300				A300/A300			
4h	VEH	REG	ARA	R+A	VEH	REG	ARA	R+A
p70 S6K	100	100	101	100	100	100	98	100
P-p70 T389	100	89*	98	88*	100	96	101	97
JNK1/2	100	98	98	99	100	99	100	99
P-JNK1/2	100	98	101	97	100	90*	90*	89*
p38	100	100	100	99	100	99	101	100
P-p38	100	108	104	104	100	104	102	104
ERK2	100	100	101	102	100	100	100	100
P-ERK1/2	100	85*	96	80*	100	94	100	94
ERBB1	100	99	100	100	100	100	99	99
P-ERBB1	100	78*	93	77*	100	95	102	85*
ERBB2	100	101	101	102	100	99	98	101
P-ERBB2	100	95	97	83*	100	96	102	96
ERBB3	100	99	100	101	100	100	101	101
P-ERBB3	100	97	99	79*	100	95	92	95
ERBB4	100	101	100	99	100	101	99	100
P-ERBB4	100	96	100	95	100	97	100	96
ERK2	100	101	101	101	100	101	101	101

HCT116 T300/T300 cells and HCT116 A300/A300 cells in 96-well plates were treated with vehicle control, aramchol (20 µM), regorafenib (0.5 µM) or the drugs combined for 4 h. Cells were fixed in place, permeabilized and subjected to in-cell immunostaining for the indicated proteins/phospho-proteins. Cells were imaged using an Odyssey infrared imager. ERK2 staining was used as an invariant loading control. The percentage alteration in expression/phosphorylation caused by the drugs was determined from three independent replicates. Values with a $p < 0.05$ were considered significant. $^{*}p < 0.05$ less than vehicle control.

Supplementary Table 4: Aramchol and regorafenib interact to activate ATM, AMPK, ULK1 and inactivate mTORC1 in HuH7 cells

HuH7 4h	VEH	REG	ARA	R+A
ATM	100	100	101	101
P-ATM S1981	100	118#	102	121#
AMPKα	100	100	100	100
P-AMPKα T172	100	123#	104	128#
mTOR	100	100	100	100
P-mTORC1 S2448	100	88*	99	80**
P-mTORC2 S2481	100	86*	100	82*
ERK2	100	100	99	100
ULK1	100	99	100	100
P-ULK1 S757	100	85*	100	80*
P-ULK1 S317	100	121#	105	123#
eIF2α	100	100	101	100
P-eIF2α S51	100	119#	101	122#
PERK	100	100	100	101
P-PERK T980	100	126#	103	131#
ERK2	100	101	101	101
AKT	100	100	100	100
P-AKT T308	100	86*	100	80*
STAT3	100	98	101	99
P-STAT3 Y705	100	91*	95	87*
STAT5	100	99	98	99
P-STAT5 Y694	100	90*	99	90*
ERK2	100	100	100	100

4h	VEH	REG	ARA	R+A
Beclin1	100	121#	99	124#
ATG5	100	120#	100	124#
ATG13	100	100	100	100
P-ATG13 S318	100	117#	103	120#
GRP78	100	121#	102	121#
CHOP	100	101	99	102
PP1	100	103	101	103
ERK2	100	100	99	99
p65 NFκB	100	101	100	100
P-p65 NFκB S536	100	87*	95	83*
c-SRC	100	100	99	99
P-c-SRC Y416	100	88*	99	86*
P-c-SRC Y527	100	107	103	112#
c-MET	100	100	101	101
P-c-MET	100	98	99	96
ERK2	100	100	100	100
CD95	100	99	100	100
FAS-L	100	97	101	95
JAK2	100	100	100	98
P-JAK2	100	95	99	95
c-KIT	100	102	101	101
P-c-KIT	100	96	100	96
ERK2	100	100	100	99

4h	VEH	REG	ARA	R+A
p70 S6K	100	99	99	99
P-p70 T389	100	95	99	93
JNK1/2	100	101	101	101
P-JNK1/2	100	99	100	97
p38	100	100	101	101
P-p38	100	96	100	103
P-ERK1/2	100	92	100	87*
ERK2	100	99	99	100
ERBB1	100	101	100	100
P-ERBB1	100	93	95	89*
ERBB2	100	100	101	100
P-ERBB2	100	97	102	102
ERBB3	100	101	101	101
P-ERBB3	100	100	100	102
ERK2	100	100	100	100
ERBB4	100	100	101	101
P-ERBB4	100	96	100	96
BCL-XL	100	91	102	88*
MCL1	100	88*	91	84*
BAX	100	105	102	105
BAK	100	103	100	103
BIM	100	100	99	104
ERK2	100	99	99	100

4h	VEH	REG	ARA	R+A
LATS1/2	100	101	100	100
P-LATS T1097	100	103	100	101
P-LATS S909	100	103	100	102
YAP	100	100	100	101
P-YAP S109	100	100	99	104
P-YAP S127	100	102	100	102
P-YAP S397	100	101	101	102
TAZ	100	100	99	100
P-TAZ S89	100	102	98	103
ERK2	100	100	99	100
PDGFRβ	100	99	100	100
P-PDGFRβ	100	92	95	89*
HSP70	100	100	100	99
HSP90	100	99	99	99
PD-L1	100	100	100	101
MHCA	100	92	97	90*
ERK2	100	100	101	101

HuH7 human adult male hepatoma cells were seeded in 96-well plates were treated with vehicle control, aramchol (20 μM), regorafenib (0.5 μM) or the drugs combined for 4 h. Cells were fixed in place, permeabilized and subjected to in-cell immunostaining for the indicated proteins/phospho-proteins. Cells were imaged using an Odyssey infrared imager. ERK2 staining was used as an invariant loading control. The percentage alteration in expression/phosphorylation caused by the drugs was determined from three independent replicates. Values with a $p < 0.05$ were considered significant. $^{\#}p < 0.05$ greater than vehicle control; $^{*}p < 0.05$ less than vehicle control; $^{**}p < 0.05$ less than regorafenib alone value.
